# Validity across four common street-crossing distraction indicators to predict pedestrian safety

**DOI:** 10.1186/s12889-024-17756-y

**Published:** 2024-01-20

**Authors:** Peishan Ning, Cifu Xie, Peixia Cheng, Li Li, David C. Schwebel, Yang Yang, Jieyi He, Jie Li, Guoqing Hu

**Affiliations:** 1 Department of Epidemiology and Health Statistics, Hunan Provincial Key Laboratory of Clinical Epidemiology, Xiangya School of Public Health, Central South University, Changsha, 410013 China; 2 Changsha Center for Disease Control and Prevention, Changsha, 410004 China; 3 Department of Child, Adolescent and Women’s Health, School of Public Health, Capital Medical University, Beijing, 100071 China; 4 Department of Psychology, University of Alabama at Birmingham, Birmingham, Alabama, 35233 USA; 5 Department of Statistics, Franklin College of Arts and Sciences, University of Georgia, Athens, GA USA; 6 National Clinical Research Center for Geriatric Disorders, Xiangya Hospital, Central South University, Changsha, 410078 China

**Keywords:** Predictive validity, Street-crossing distraction, Indicator, Pedestrian, Safety

## Abstract

**Background:**

Multiple distraction indicators have been applied to measure street-crossing distraction but their validities in predicting pedestrian safety are poorly understood.

**Methods:**

Based on a video-based observational study, we compared the validity of four commonly used distraction indicators (total duration of distraction while crossing a street, proportion of distracted time over total street-crossing time, duration of the longest distraction time, and total number of distractions) in predicting three pedestrian safety outcomes (near-crash incidence, frequency of looking left and right, and speed crossing the street) across three types of distraction (mobile phone use, talking to other pedestrians, eating/drinking/smoking). Change in Harrell’s C statistic was calculated to assess the validity of each distraction indicator based on multivariable regression models including only covariates and including both covariates and the distraction indicator.

**Results:**

Heterogeneous capacities in predicting the three safety outcomes across the four distraction indicators were observed: 1) duration of the longest distraction time was most predictive for the occurrence of near-crashes and looks left and right among pedestrians with all three types of distraction combined and talking with other pedestrians (Harrell’s C statistic changes ranged from 0.0310 to 0.0335, *P* < 0.05), and for the occurrence of near-crashes for pedestrians involving mobile phone use (Harrell’s C statistic change: 0.0053); 2) total duration of distraction was most predictive for speed crossing the street among pedestrians with the combination and each of the three types of distraction (Harrell’s C statistic changes ranged from 0.0037 to 0.0111, *P* < 0.05), frequency of looking left and right among pedestrians distracted by mobile phone use (Harrell’s C statistic change: 0.0115), and the occurrence of near-crash among pedestrians eating, drinking, or smoking (Harrell’s C statistic change: 0.0119); and 3) the total number of distractions was the most predictive indicator of frequency of looking left and right among pedestrians eating, drinking, or smoking (Harrell’s C statistic change: 0.0013). Sensitivity analyses showed the results were robust to change in grouping criteria of the four distraction indicators.

**Conclusions:**

Future research should consider the pedestrian safety outcomes and type of distractions to select the best distraction indicator.

**Supplementary Information:**

The online version contains supplementary material available at 10.1186/s12889-024-17756-y.

## Background

As mobile phone use has increased worldwide, distracted pedestrian behavior while crossing the street has also become more and more common. The reported prevalence of street-crossing distraction ranges from 6.4% to 43.2% among pedestrians globally, with rates varying across location and time period [1–3]. The number of injuries reported to be related to crossing streets while being distracted has also risen dramatically in many countries. For example, the number of pedestrians in the United States known to be killed by distraction from portable electronic devices, talking, or eating increased from 97 to 276 between 2010 and 2019 [4].

To develop strategies for prevention of distracted pedestrian behavior and related crashes and injuries, valid epidemiological indicators are important. These indicators should quantify the extent of street-crossing distraction and allow researchers to examine associations between pedestrian distraction and injury outcomes. However, the current literature lacks validation of pedestrian-specific distraction indicators [3].

The existing literature does include multiple indicators that are currently used in distracted driving research [5–7]; they are based on the type, duration, and frequency of street-crossing distraction. Several indicators have been empirically demonstrated to exhibit significant correlations with driving performance and the likelihood of crashes or near-crashes in naturalistic driving studies [6, 7]. However, the comparative validity of these indicators has not been examined through rigorous empirical study. It remains unclear if and how these distraction indicators may correlate with pedestrian safety outcomes or whether a single indicator is most predictive of risk.

We conducted a large video-based observational study to compare the validity of four common street-crossing distraction indicators for predicting pedestrian safety. Specially, we tested three hypotheses: (a) longer distraction time leads to near-crashes and failure to look at traffic and therefore is a predictive indicator of distraction for those two pedestrian safety outcomes, (b) total distraction time causes people to walk more slowly and therefore is a predictive distraction indicator for speed crossing the street, and (c) a larger number of distractions causes pedestrian to look less at traffic and is a predictive distraction indicator for frequency of looking left and right.

## Methods

### Study design

**Fig. 1:**
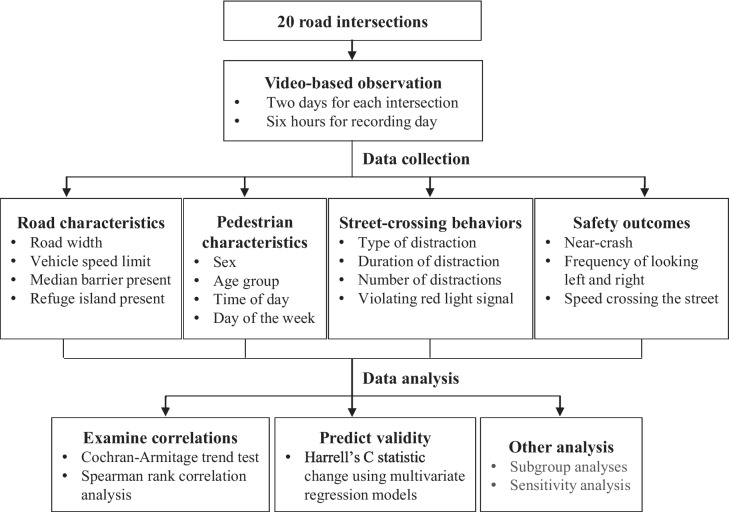
Flowchart of the study design. Note: The definitions or classifications of collected data were described in the street-crossing distraction indicators, pedestrian safety outcomes, and covariates section

Data collected for a large video-based observational study were used to conduct a secondary comparative analysis assessing the validity of four common distraction indicators to predict pedestrian safety (Fig. 1).

### Data source

Data were collected in 2019 at 20 “╋” type road intersections in Changsha, China (Trial registration number: ChiCTR1900023791) [8]. Overall, 240 h of videotaped street-crossing behaviors were collected. Detailed information of the study design appears in Supplemental Appendix A.

Because pedestrians younger than age 20 years accounted for less than 10% of all pedestrians and it is difficult to obtain informed consent for research from adult caregivers of young children in field observations [9], our analysis focused on adult pedestrians appearing to be aged 20 years and older.

### Street-crossing distraction indicators

As defined below, four commonly-used indicators of street-crossing distraction were considered [5–7, 10]. Researchers flagged the videos to identify the starting and ending time of each distraction that a pedestrian displayed while crossing the street, allowing us to determine the full temporal duration of distraction.

- *Total duration of distraction while crossing a street*: Total duration of time the pedestrian was distracted while crossing the street, in seconds.
- *Proportion of distracted time over total street-crossing time:* Proportion of distracted time over total duration of time crossing the street.
- *Duration of the longest distraction time*: Duration of the longest distraction the pedestrian displays while crossing the street, in seconds.
- *Total number of distractions*: Number of distractions the pedestrian displays while crossing the street.

We also considered the type of distraction, which was coded as mobile phone use, talking to other pedestrians, eating, drinking, or smoking. The latter three categories were merged into a single category due to small sample sizes.

In total, 25,436 adult pedestrians were observed. Of those, 34.3% (*n* = 8,729) were undistracted while crossing, 32.8% (*n* = 8,347) displayed one type of distraction, and 1.5% (*n* = 384) displayed multiple types of distraction (Supplemental Table B1).

The indicators *a*-*c* were categorized into five groups in fitting multivariable models based on the quartiles of sample distributions (no distraction = 0; *P*_0.1_ ~ *P*_25_ = 1; *P*_25.1_ ~ *P*_50_ = 2; *P*_50.1_ ~ *P*_75_ = 3; > *P*_75_ = 4) by type of distraction; the indicator *d* was categorized into four groups based on distribution (no distraction = 0; distracted one time = 1; two times = 2; three or more times = 3).

### Pedestrian safety outcomes

Following previous research, we considered three pedestrian safety outcome measures:

- *Near-crash incidence* [11]: A near-crash event was defined as any circumstance that required a rapid, evasive maneuver by either the pedestrian or any motor or non-motor vehicle (or both) to avoid a pedestrian-vehicle crash. Evasive maneuvers for pedestrians included physically-obvious changes in direction, stopping, or running forward [11]. The near-crash outcome was transformed into a binary variable, having a near-crash event or not. Near-crash incidence was calculated as “the number of pedestrians encountering a near-crash event / total number of observed pedestrians × 100%”. We observed no actual crashes.
- *Frequency of looking left and right* [12, 13]: Frequency of looking left and right was defined as the number times a pedestrian distinctly moved their head left or right while crossing the street. A much low frequency of looking left and right generally indicates a longer distraction period, as distracted pedestrians divert their gaze away from the road and experience diminished awareness of their surroundings. This behavior reduces safety while crossing the street [13, 14].
- *Speed crossing the street* [13]: Speed crossing the street was defined as the average distance a pedestrian walked per 10 s while crossing the street. Lower speeds while crossing the street generally lead to extended time duration on the road, increased exposure to traffic and therefore an elevated risk for crashes [14, 15].

Data for the three safety measures were manually transcribed from the videos by trained researchers (Supplemental Appendix A).

### Covariates

Based on their reported relevance in previous research [16, 17], we included the following nine covariates in analyses: sex (male vs. female), estimated age group (20–39 years, 40–59 years, or ≥ 60 years), time of day (morning vs. afternoon), day of the week (weekday vs. weekend), violating red light signal (yes vs. no), road width (< 22 m, 22–31 m, and ≥ 32 m, based on the tertiles of *P*_33.3_ and *P*_66.7_), vehicle speed limit displayed prominently at intersection, median barrier present on the road, and refuge island present. Descriptive data on the covariates appear in Supplemental Tables B1 and B2.

### Reliability of data transcription

Pilot research demonstrated high reliability between manually transcribed data of videos and data collected through face-to-face interviews with 300 pedestrians for sex (99.7%) and age group (93.0%). Re-transcription of 20% randomly selected videos (48 of 240 h) showed high reproducibility consistency (93.1%) for all study variables combined.

### Statistical analysis

The Cochran-Armitage trend test and Spearman rank correlation analysis were used to examine correlations between the three safety outcome measures and between the four distraction indicators.

Following previous research [18, 19], we adopted the change of Harrell’s C statistic in multivariate regression models to assess the predictive validity of each distraction indicator. Models were constructed including the nine covariates mentioned above versus including both the nine covariates and the street-crossing distraction indicator. The link functions of the multivariate regression models were linear functions for average speed crossing the street, quasi-Poisson functions for frequency of looking left and right (offset by total duration of time crossing the street), and logistic functions for near-crash events. Harrell’s C statistic is essentially a rank-correlation measure that represents the capacity of discrimination [20]. Harrell’s C statistic is also referred to as the estimated area under the Receiver Operating Characteristics curve quantified for binary outcomes [21]. 95% confidence intervals (95% CI) of Harrell’s C statistic change were estimated using bootstrapped standard errors with 1000 replications [22, 23]. Detailed groupings of the four distraction indicators are shown in Supplemental Table B3.

Subgroup analyses were performed by type of distraction. Due to small sample sizes, analyses for multiple types of street-crossing distractions were omitted. The variance inflation factor for all independent variables in all fitted models ranged from 1.00 to 3.17, suggesting the absence of substantial collinearity between regressors.

We performed sensitivity analysis to assess the stability of results across different groupings of the four distraction indicators. To do this, we used median, tertiles, and quintiles respectively to classify pedestrians for distraction indicators *a-c* and used different combination schemes to group pedestrians for distraction indicator *d*, as shown in Supplemental Table B4.

All statistical analyses were performed using SAS 9.4 (SAS Institute) and R 4.1.2. A *p*-value of less than 0.05 was considered statistically significant.

## Results

### Sample characteristics

**Fig. 2:**
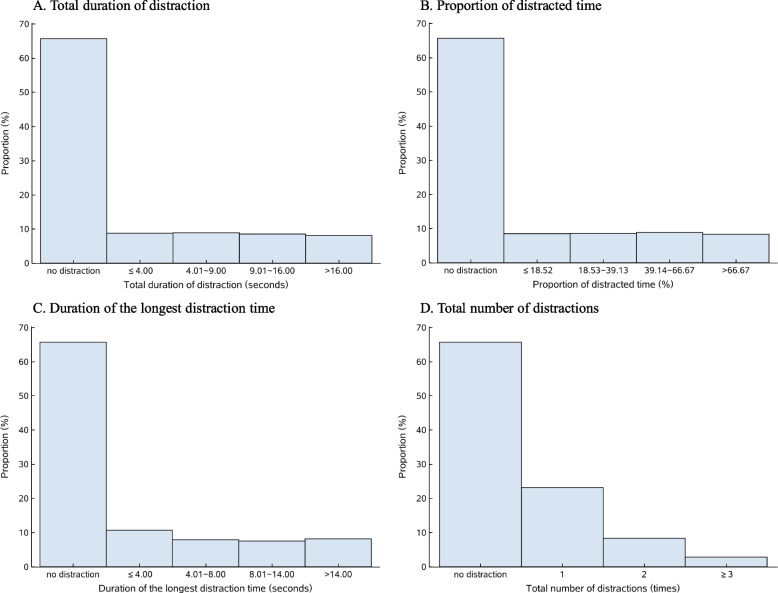
Distribution of the four distraction indicators among 25,436 videotaped pedestrians

**Fig. 3:**
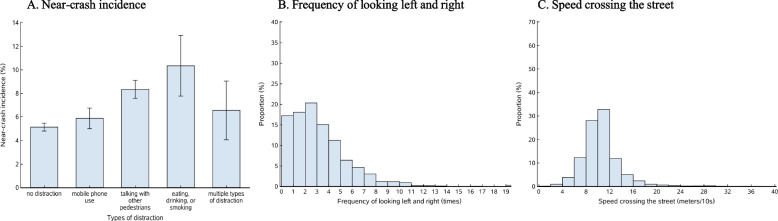
Distribution of the three pedestrian safety measures among 25,436 videotaped pedestrians

In total, we observed street-crossing by 25,436 pedestrians estimated to be aged 20 years and older, including 10,886 males (42.8%) and 14,550 females (57.2%). 15,111, 7,741, and 2,584 pedestrians were estimated to be aged 20–39 years (59.4%), aged 40–59 years (30.4%), and aged 60 years and older (10.2%), respectively (Supplemental Table B1). Of them, 2,814 (19.6%), 4,991 (11.0%), and 542 (2.1%) were distracted by mobile phone use, talking with other pedestrians, and eating, drinking, or smoking, respectively. Sample data for all four distraction indicators and two of the pedestrian safety measures demonstrated positively skewed distributions (Figs. 2 and 3).

### Correlations between distraction indicators and pedestrian safety measures

The four distraction indicators were all slightly or moderately correlated with the three pedestrian safety measures (*p* < 0.05, Supplemental Figs. B1-B12), except for insignificant correlations between (a) speed crossing the street for all walking distractions combined with the proportion of distracted time and the total number of distractions (*r*_s_ = -0.01, *p* = 0.32, and *r*_s_ = -0.01, *p* = 0.12, respectively) (Supplemental Fig. B3), (b) total number of distractions with near-crash incidence for mobile phone use (*Z* = 1.51, *p* = 0.13, Supplemental Fig. B4), and (c) all four distraction indicators with frequency of looking left and right for eating, drinking, or smoking (*p* > 0.05, Supplemental Fig. B11).

### Predictive validity of the four distraction indicators

#### All distractions

**Fig. 4:**
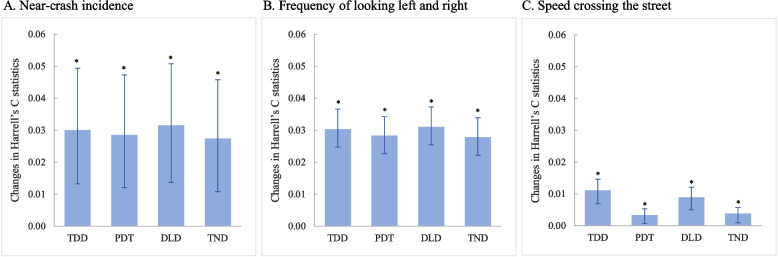
Validity of the four distraction indicators for street-crossing safety among all distracted pedestrians. Abbreviations: TDD – total duration of distraction while crossing a street, PDT – proportion of distracted time over total street-crossing time, DLD – duration of the longest distraction time, and TND – total number of distractions. The TDD, PDT, and DLD were grouped by quartile; TND were grouped as no distraction, distracted one time, distracted two times, and distracted three or more times (Supplemental Table B4)

For all distractions combined, the four distraction indicators were significantly predictive for all three pedestrian safety outcomes (*p* < 0.05, Fig. 4 and Supplemental Table B5). All four distraction indicators showed the largest predictive validity for near-crash incidence (Harrell’s C statistic change: 0.0274–0.0315) and the smallest predictive validity for speed crossing the street (Harrell’s C statistic change: 0.0033–0.0111).

Of the four distraction indicators, duration of the longest distraction time was most predictive for near-crash incidence (Harrell’s C statistic change: 0.0315, 95% CI: 0.0137–0.0508) and frequency of looking left and right (Harrell’s C statistic change: 0.0310, 95% CI: 0.0254–0.0373), while total duration of distraction was most predictive for speed crossing the street (Harrell’s C statistic change: 0.0111, 95% CI: 0.0068–0.0146) (Fig. 4 and Supplemental Table B5).

Because the predictive validity of the four distraction indicators varied across the type of distraction, we considered subgroup analysis by type of distraction next.

#### Mobile phone use

**Fig. 5:**
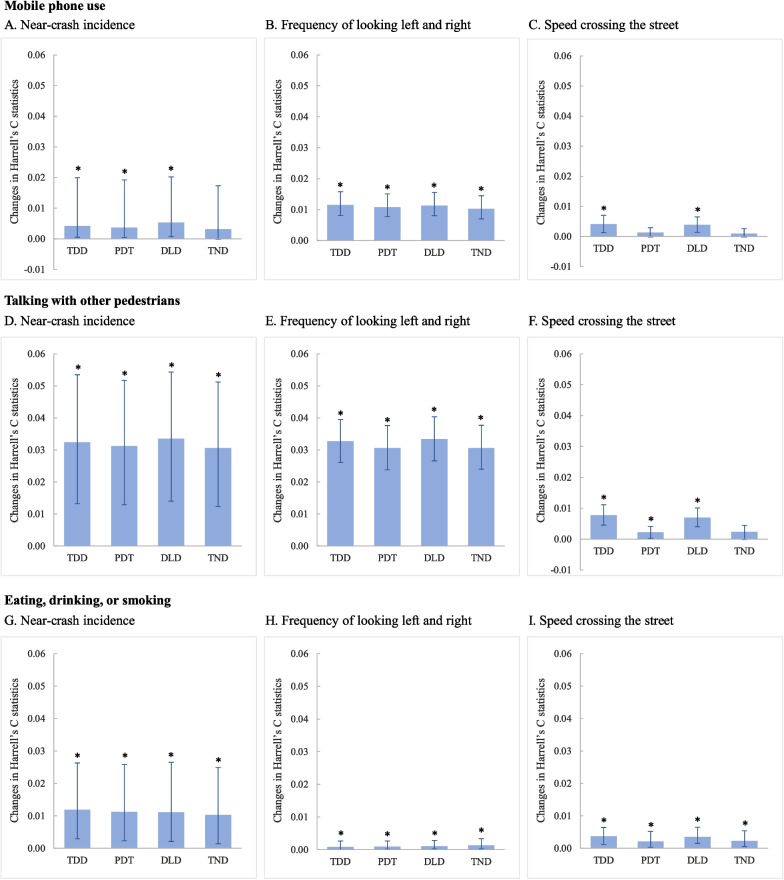
Validity of the four distraction indicators for street-crossing safety by type of distraction. Abbreviations: TDD – total duration of distraction while crossing a street, PDT – proportion of distracted time over total street-crossing time, DLD – duration of the longest distraction time, and TND – total number of distractions. The TDD, PDT, and DLD were grouped by quartile; TND were grouped as no distraction, distracted one time, distracted two times, and distracted three or more times (Supplemental Table B4)

When focused only on mobile phone use instead of all distractions, the four street-crossing distraction indicators were all significantly predictive of the three pedestrian safety outcomes (*p* < 0.05, Fig. 5A-C and Supplemental Table B6), except for the total number of distractions predicting incidence of near-crash and speed crossing the street, and duration of the longest distraction time for predicting speed crossing the street (*p* > 0.05). All four distraction indicators displayed the greatest predictive validity for frequency of looking left and right (Harrell’s C statistic change: 0.0103–0.0115) but the least predictive validity for speed crossing the street (Harrell’s C statistic change: 0.0010–0.0041).

Of the four distraction indicators, duration of the longest distraction time by mobile phone use was most predictive of near-crashes (Harrell’s C statistic change: 0.0053, 95% CI: 0.0007–0.0202), while total duration of distraction by mobile phone was most predictive of frequency of looking left and right and speed crossing the street (Harrell’s C statistic change: 0.0115, 95% CI: 0.0081–0.0158; and 0.0041, 95% CI: 0.0013–0.0070) (Fig. 5A-C and Supplemental Table B6).

#### Talking with other pedestrians

When focused only on distraction talking with other pedestrians, the four street-crossing distraction indicators were all significantly predictive of the three pedestrian safety measures (*p* < 0.05, Fig. 5D-F and Supplemental Table B7), except for the total number of distractions for predicting speed crossing the street (*p* > 0.05). All four distraction indicators showed the largest predictive validity for near-crash incidence (Harrell’s C statistic change: 0.0306–0.0335) and the least predictive validity for predicting speed crossing the street (Harrell’s C statistic change: 0.0022–0.0077).

Duration of the longest distraction time was most predictive of near-crash incidence (Harrell’s C statistic change: 0.0335, 95% CI: 0.0140–0.0543) and frequency of looking left and right (Harrell’s C statistic change: 0.0334, 95% CI: 0.0266–0.0403), while total duration of distraction talking to other pedestrians offered the best predictive validity of speed crossing the street (Harrell’s C statistic change: 0.0077, 95% CI: 0.0045–0.0111) (Fig. 5D-F and Supplemental Table B7).

#### Eating, drinking, or smoking

When distraction was limited to eating, drinking, or smoking, the four street-crossing distraction indicators were all significantly predictive of the three pedestrian safety outcomes (*p* < 0.05, Fig. 5G-I and Supplemental Table B8). All four distraction indicators displayed the largest predictive validity for near-crash incidence (Harrell’s C statistic change: 0.0103–0.0119) and the least predictive validity of frequency of looking left and right (Harrell’s C statistic change: 0.0008–0.0013).

Of the four distraction indicators, total duration of distraction had the largest predictive validity for near-crash incidence (Harrell’s C statistic changes: 0.0119, 95% CI: 0.0029–0.0263) and speed crossing the street (Harrell’s C statistic change: 0.0037, 95% CI: 0.0012–0.0064), while total number of distractions had the largest predictive validity of frequency of looking left and right (Harrell’s C statistic change: 0.0013, 95% CI: 0.0002–0.0034) (Fig. 5G-I and Supplemental Table B8).

### Sensitivity analyses

Sensitivity analyses were conducted by changing the groupings of the four distraction indicators and showed robust results to the changes (Supplemental Tables B5-B8).

## Discussion

### Primary findings

Using observational strategies with a large sample of data recorded at Chinese intersections, this study compared the predictive validity of four commonly used street-crossing distraction indicators for three street-crossing safety outcomes. All four distraction indicators – total duration of distraction, proportion of distracted time, duration of the longest distraction time, and total number of distractions – were generally predictive of the three pedestrian safety outcomes – near-crash incidence, frequency of looking left and right, and speed crossing the street. However, the indicators showed heterogeneous predictive capacities for different pedestrian safety outcomes and type of distraction (Table 1).

**Table 1:** Street-crossing distraction indicators most predictive for three street-crossing safety outcomes

**Type of distraction**	**Safety outcome**	**TDD**	**PDT**	**DLD**	**TND**
All distractions	Near-crash incidence			√	
	Frequency of looking left and right			√	
	Speed crossing the street	√			
Mobile phone use	Near-crash incidence			√	
	Frequency of looking left and right	√			
	Speed crossing the street	√			
Talking with other pedestrians	Near-crash incidence			√	
	Frequency of looking left and right			√	
	Speed crossing the street	√			
Eating, drinking, or smoking	Near-crash incidence	√			
	Frequency of looking left and right				√
	Speed crossing the street	√			

The TDD, PDT, and DLD were grouped by quartile; TND were grouped as no distraction, distracted one time, distracted two times, and distracted three or more times (Supplemental Table B4)

*Abbreviations*: *TDD* Total duration of distraction while crossing a street, *PDT* Proportion of distracted time over total street-crossing time, *DLD* Duration of the longest distraction time, *TND* Total number of distractions

Duration of the longest distraction time was most predictive of the occurrence of near-crashes and the frequency of looking left and right among pedestrians with all types of distraction combined and for talking with other pedestrians, and for the occurrence of near-crashes among pedestrians distracted by mobile phone use. Total duration of distraction was the best indicator for predicting (a) speed crossing the street outcomes among pedestrians with all three types of distraction, (b) frequency of looking left and right among pedestrians distracted by mobile phone use, and (c) near-crash incidence among pedestrians distracted by eating, drinking, or smoking. Finally, total number of distractions was the most predictive indicator of frequency of looking left and right among pedestrians distracted by eating, drinking, or smoking.

### Interpretation of findings

As distracted pedestrian behavior increases globally and leads to increasing pedestrian injury and death rates, researchers will benefit from an understanding of the predictive validity of street-crossing distraction indicators for street-crossing safety. Previous research in this domain is scarce; just one published study in the driving literature examined associations between crashes or near-crashes with two driving distraction indicators, the driver’s single longest glance off the roadway and the total duration of eyes off the roadway [6]. That study found that the risk of crash increased with the duration of the single longest glance in all secondary tasks and wireless secondary task engagement, but it focused only on newly licensed teenage drivers and relied on a single safety outcome measure.

The heterogeneous predictive validities of the four distraction indicators we discovered are logical in theory, as the indicators measure different aspects of distraction involving different cognitive and perceptual distraction processes [11]. For example, talking with other pedestrians is likely to significantly affect cognitive and aural attention but only minimally affect visual attention. Eating, drinking, and smoking may influence kinetic movements and perhaps have some cognitive impact, but their impact on visual or aural attention could be minimal [5]. Mobile phone use distracts pedestrians in multiple ways simultaneously, having cognitive, kinetic, visual and sometimes aural components that can impact pedestrian safety [11].

One could also consider our results from the perspective of pedestrian outcomes. Frequency of looking left and right is largely a visual task, though it impacts efficient cognitive processing of the perceived traffic environment. It was best predicted by the duration of the longest distraction time and by the total duration of distraction for the more visually distracting situations, including mobile phone use and talking with other pedestrians. While frequency of looking left and right was better predicted by the total number of distractions for eating, drinking, or smoking that involves fewer visual distractions. Speed crossing the street is driven primarily by cognitive distraction, which impacts gait speed [24, 25]. It was best predicted by the total number of distractions.

Our findings provide valuable evidence to support targeted selection of street-crossing distraction indicators in scientific research and prevention practice. The results will help guide researchers and practitioners to select optimal street-crossing distraction indicators that quantify the extent of street-crossing distraction in particular circumstances and for particular goals. With greater precision in the design of both observational and experimental research, researchers can better develop and evaluate effective intervention programs to reduce distracted pedestrian behavior and resultant pedestrian injuries and deaths.

### Limitations

This study has several limitations. First, despite the large sample size, we did not capture any pedestrian-related crashes or injuries to use as outcome measures. Instead, we relied on common proxy measures like near-crash events [26] that may not perfectly represent prediction of crash or injury outcomes. Second, our analysis was based on videos taken at 20 “╋” type road intersections and among pedestrians appearing to be aged 20 years and older. Considering the highly similar road traffic environments and pedestrian safety culture across China, results would likely generalize to other Chinese locations but may not generalize to other countries or cultures, to mid-block crossing locations, or to pedestrian behavior by children or adolescents.

Third, we relied on observational coding strategies that can generate bias based on researcher judgment. However, such methodologies are widely used [16, 17] and we employed standard strategies to establish reliability and validity of our data. Fourth, a lower frequency of looking left and right and a slower speed crossing the street generally indicate reduced surroundings awareness, increase exposure to traffic, and consequently threaten pedestrian safety. However, there are some exceptions, like stopping in the middle of the crosswalk to let a car pass while crossing a wide street. To control for these exceptional circumstances, we included red light signal violation in multivariable models as a covariate. Last, due to absence of relevant injury data (i.e., injury morbidity and mortality), our recommendations should be used with caution when using fatal and non-fatal pedestrian injuries as safety outcomes. Further studies are needed to generate relevant evidence.

## Conclusion

In conclusion, four common distraction indicators showed different validity in predicting three street-crossing safety outcomes of pedestrians. To improve scientific rigor in future research and practice initiatives, we recommend selection of street-crossing distraction indicators that consider the pedestrian safety outcomes of interest and the types of walking distraction to be studied.

### Supplementary Information

**Additional file 1: Appendix A.** Brief description of the video-based observational study in Changsha, China. **Fig. A1.** Geographic location of 20 road intersections for video-based observations in Changsha city, China. **Fig. A2.** Placement of cameras for video-based observation at road intersections. **Appendix B.** Appendix Tables and Figures. **Table B1.** Sample characteristics of pedestrians at 20 road intersections in Changsha, China collected between June 29 and July 21, 2019. **Table B2.** Basic road characteristics of the 20 included road intersections in Changsha, China. **Table B3.** Description of the grouping of the four pedestrian distraction indicators for primary analysis, by type of distraction. **Table B4.** Description of the grouping of four distraction indicators by type of distraction for sensitivity analysis. **Table B5.** Sensitivity analyses for discriminant validity of the four distraction indicators by alternating the classification of distraction indicators, all walking distractions combined. **Table B6.** Sensitivity analyses for discriminant validity of four distraction indicators by alternating the grouping of distraction indicator, mobile phone use. **Table B7.** Sensitivity analyses for discriminant validity of four distraction indicators by alternating the grouping of distraction indicator, talking with other pedestrians. **Table B8.** Sensitivity analyses for discriminant validity of four distraction indicators by changing the grouping of distraction indicator, eating, drinking, or smoking. **Fig. B1.** Linear graph showing the associations between the four distraction indicators and near-crash incidence, all walking distractions combined. **Fig. B2.** Linear graph showing the associations between the four distraction indicators and frequency of looking left and right, all walking distractions combined. **Fig. B3.** Linear graph showing the associations between the four distraction indicators and speed crossing the street, all walking distractions combined. **Fig. B4.** Linear graph showing the associations between the four distraction indicators and near-crash incidence, mobile phone use. **Fig. B5.** Linear graph showing the associations between the four distraction indicators and frequency of looking left and right, mobile phone use. **Fig. B6.** Linear graph showing the associations between the four distraction indicators and speed crossing the street, mobile phone use. **Fig. B7.** Linear graph showing the associations between the four distraction indicators and near-crash incidence, talking with other pedestrians. **Fig. B8.** Linear graph showing the associations between the four distraction indicators and frequency of looking left and right, talking with other pedestrians. **Fig. B9.** Linear graph showing the associations between the four distraction indicators and speed crossing the street, talking with other pedestrians. **Fig. B10.** Linear graph showing the associations between the four distraction indicators and speed near-crash incidence, eating, drinking, or smoking. **Fig. B11.** Linear graph showing the associations between the four distraction indicators and frequency of looking left and right, eating, drinking, or smoking. **Fig. B12.** Linear graph showing the four distraction indicators and speed crossing the street, eating, drinking, or smoking.

## Data Availability

The datasets used and/or analysed during the current study are available from the corresponding author on reasonable request.

## Abbreviations

TDD: Total duration of distraction while crossing a street
PDT: Proportion of distracted time over total street-crossing time
DLD: Duration of the longest distraction time
TND: Total number of distractions
