# Supplementary material for: Validity across four common street-crossing distraction indicators to predict pedestrian safety
Source: BMC Public Health. 2024 Jan 20;24:241. doi: 10.1186/s12889-024-17756-y (PMC10799455; doi:10.1186/s12889-024-17756-y)
Supplement: Supplementary file 1 — Additional file 1: Appendix A. Brief description of the video-based observational study in Changsha, China. Fig. A1. Geographic location of 20 road intersections for video-based observations in Changsha city, China. Fig. A2. Placement of cameras for video-based observation at road intersections. Appendix B. Appendix Tables and Figures. Table B1. Sample characteristics of pedestrians at 20 road intersections in Changsha, China collected between June 29 and July 21, 2019. Table B2. Basic road characteristics of the 20 included road intersections in Changsha, China. Table B3. Description of the grouping of the four pedestrian distraction indicators for primary analysis, by type of distraction. Table B4. Description of the grouping of four distraction indicators by type of distraction for sensitivity analysis. Table B5. Sensitivity analyses for discriminant validity of the four distraction indicators by alternating the classification of distraction indicators, all walking distractions combined. Table B6. Sensitivity analyses for discriminant validity of four distraction indicators by alternating the grouping of distraction indicator, mobile phone use. Table B7. Sensitivity analyses for discriminant validity of four distraction indicators by alternating the grouping of distraction indicator, talking with other pedestrians. Table B8. Sensitivity analyses for discriminant validity of four distraction indicators by changing the grouping of distraction indicator, eating, drinking, or smoking. Fig. B1. Linear graph showing the associations between the four distraction indicators and near-crash incidence, all walking distractions combined. Fig. B2. Linear graph showing the associations between the four distraction indicators and frequency of looking left and right, all walking distractions combined. Fig. B3. Linear graph showing the associations between the four distraction indicators and speed crossing the street, all walking distractions combined. Fig. B4. Linear graph showi [file 12889_2024_17756_MOESM1_ESM.docx]

**Validity across four common street-crossing distraction indicators to predict pedestrian safety: supplementary material**

**Contents**

[**Appendix A. Brief description of the video-based observational study in Changsha, China** 1](#_Toc144105581)

[**Fig. A1** Geographic location of 20 road intersections for video-based observations in Changsha city, China 1](#_Toc144105582)

[**Fig. A2** Placement of cameras for video-based observation at road intersections 2](#_Toc144105583)

[**Appendix B. Appendix Tables and Figures** 3](#_Toc144105584)

[**Table B1** Sample characteristics of pedestrians at 20 road intersections in Changsha, China collected between June 29 and July 21, 2019 3](#_Toc144105585)

[**Table B2** Basic road characteristics of the 20 included road intersections in Changsha, China 4](#_Toc144105586)

[**Table B3** Description of the grouping of the four pedestrian distraction indicators for primary analysis, by type of distraction 5](#_Toc144105587)

[**Table B4** Description of the grouping of four distraction indicators by type of distraction for sensitivity analysis 6](#_Toc144105588)

[**Table B5** Sensitivity analyses for discriminant validity of the four distraction indicators by alternating the classification of distraction indicators, all walking distractions combined 8](#_Toc144105589)

[**Table B6** Sensitivity analyses for discriminant validity of four distraction indicators by alternating the grouping of distraction indicator, mobile phone use 11](#_Toc144105590)

[**Table B7** Sensitivity analyses for discriminant validity of four distraction indicators by alternating the grouping of distraction indicator, talking with other pedestrians 14](#_Toc144105591)

[**Table B8** Sensitivity analyses for discriminant validity of four distraction indicators by changing the grouping of distraction indicator, eating, drinking, or smoking 17](#_Toc144105592)

[**Fig. B1** Linear graph showing the associations between the four distraction indicators and near-crash incidence, all walking distractions combined 20](#_Toc144105593)

[**Fig. B2** Linear graph showing the associations between the four distraction indicators and frequency of looking left and right, all walking distractions combined 21](#_Toc144105594)

[**Fig. B3** Linear graph showing the associations between the four distraction indicators and speed crossing the street, all walking distractions combined 22](#_Toc144105595)

[**Fig. B4** Linear graph showing the associations between the four distraction indicators and near-crash incidence, mobile phone use 23](#_Toc144105596)

[**Fig. B5** Linear graph showing the associations between the four distraction indicators and frequency of looking left and right, mobile phone use 24](#_Toc144105597)

[**Fig. B6** Linear graph showing the associations between the four distraction indicators and speed crossing the street, mobile phone use 25](#_Toc144105598)

[**Fig. B7** Linear graph showing the associations between the four distraction indicators and near-crash incidence, talking with other pedestrians 26](#_Toc144105599)

[**Fig. B8** Linear graph showing the associations between the four distraction indicators and frequency of looking left and right, talking with other pedestrians 27](#_Toc144105600)

[**Fig. B9** Linear graph showing the associations between the four distraction indicators and speed crossing the street, talking with other pedestrians 28](#_Toc144105601)

[**Fig. B10** Linear graph showing the associations between the four distraction indicators and speed near-crash incidence, eating, drinking, or smoking 29](#_Toc144105602)

[**Fig. B11** Linear graph showing the associations between the four distraction indicators and frequency of looking left and right, eating, drinking, or smoking 30](#_Toc144105603)

[**Fig. B12** Linear graph showing the four distraction indicators and speed crossing the street, eating, drinking, or smoking 31](#_Toc144105604)

**Appendix A. Brief description of the video-based observational study in Changsha, China**

**Study site**

The study adopted multi-stage random sampling to determine observation sites in Changsha, China. In the first step, we divided Changsha into 412 square-shaped geographic zones (1.9 kilometers ×1.9 kilometers). An eligible zone was defined as having at least two "╋” type road intersections. In total, 261 (63%) of the 412 square zones met this criterion. Second, we randomly chose ten square zones from the 261 eligible zones and then selected two "╋” type road intersections within each of those ten zones at random. These steps led, therefore, to identification of 20 randomly selected road intersections for observation (Fig. A1). All sampling sequences were performed using SAS 9.2 software.


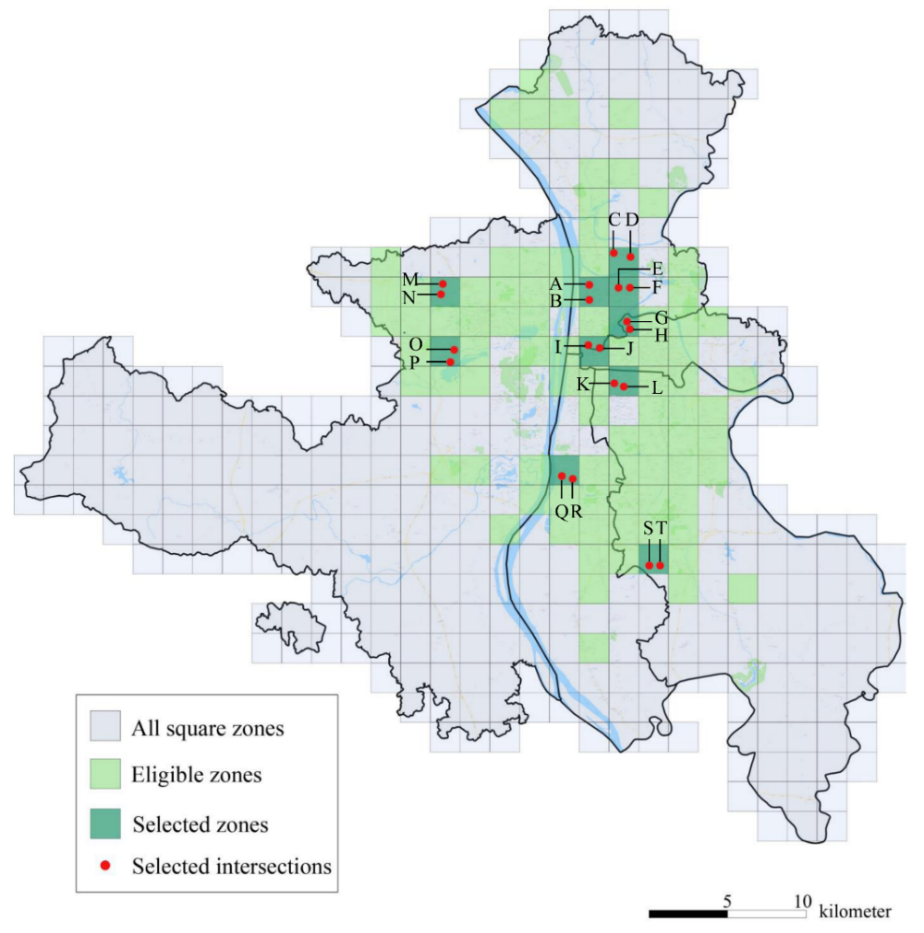


**Fig. A1** Geographic location of 20 road intersections for video-based observations in Changsha city, China

Note: Letters A-T represent the 20 selected “╋” type road intersections.

**Data collection**

The video-based observational study was conducted prior to the global COVID-19 pandemic, from June 29 to July 21, 2019. Field observations were recorded using smartphone-based high-definition cameras (REDMI NOTE3, 1080p HD) placed at each intersection site to capture crosswalk traffic lights, vehicle traffic lights, and street-crossing behavior of all passing pedestrians. Fig. A2 shows the placement of cameras at each intersection.

For each selected intersection, we recorded traffic on two days, one weekday and one weekend. On each recording day, we videotaped traffic for six hours, three peak hours (7:30-9:00 and 17:00-18:30 for weekdays; 8:30-10:00 and 17:00-18:30 for weekend) and three off-peak hours (9:00-11:00 and 16:30-17:00 for weekdays; 10:00-12:00 and 16:30-17:00 for weekend). In total, therefore, we recorded traffic for 240 hours: 20 intersections/observation × two days/intersection (weekday and weekend) × six hours/day.

**
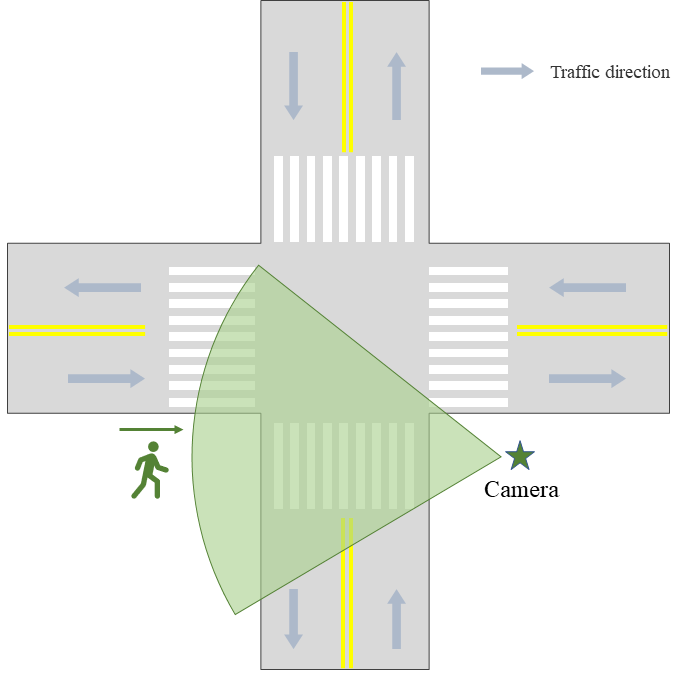
**

**Fig. A2** Placement of cameras for video-based observation at road intersections

**Appendix B. Appendix Tables and Figures**

**Table B1** Sample characteristics of pedestrians at 20 road intersections in Changsha, China collected between June 29 and July 21, 2019

| **Variable** | **Number** | **Proportion (%)** |
| --- | --- | --- |
| **Total** | 25436 | 100.0 |
| **Sex** |  |  |
| Male | 10886 | 42.8 |
| Female | 14550 | 57.2 |
| **Age group** |  |  |
| 20-39 years | 15111 | 59.4 |
| 40-59 years | 7741 | 30.4 |
| ≥60 years | 2584 | 10.2 |
| **Time of day** |  |  |
| Morning | 13672 | 53.8 |
| Afternoon | 11764 | 46.2 |
| **Time of week** |  |  |
| Weekday | 14217 | 55.9 |
| Weekend | 11219 | 44.1 |
| **Violating red light signal** |  |  |
| Yes | 14075 | 55.33 |
| No | 11361 | 44.67 |
| **Distraction while walking** |  |  |
| Yes | 8729 | 34.3 |
| Mobile phone use | 2814 | 11.1 |
| Talking with other pedestrians | 4991 | 19.6 |
| Eating, drinking, or smoking | 542 | 2.1 |
| Multiple distractions | 382 | 1.5 |
| No | 16707 | 65.7 |

Note: Multiple distractions refer to two or more simultaneous types of distraction.

**Table B2** Basic road characteristics of the 20 included road intersections in Changsha, China

| **Road characteristic** | **Number** | **Proportion (%)** |
| --- | --- | --- |
| **Total** | 20 | 100.0 |
| **Road width** |  |  |
| <22 meters | 6 | 30.0 |
| 22-31 meters | 7 | 35.0 |
| ≥32 meters | 7 | 35.0 |
| **Prominent vehicle speed limit sign** |  |  |
| Yes | 8 | 40.0 |
| No | 12 | 60.0 |
| **Median barrier** |  |  |
| Yes | 10 | 50.0 |
| No | 10 | 50.0 |
| **Refuge island** |  |  |
| Yes | 2 | 10.0 |
| No | 18 | 90.0 |

**Table B3** Description of the grouping of the four pedestrian distraction indicators for primary analysis, by type of distraction

| **Distraction indicator** | **Assignment** |
| --- | --- |
| **For all distraction** |  |
| Total duration of distraction | no distraction=0; 0.01~4.00 seconds=1; 4.01~9.00 seconds=2; 9.01~16.00 seconds=3; >16.00 seconds=4 |
| Proportion of distracted time | no distraction=0; 0.01%~18.52%=1; 18.53%~39.13%=2; 39.14%~66.67%=3; >66.67%=4 |
| Duration of the longest distraction time | no distraction=0; 0.01~4.00 seconds=1; 4.01~8.00 seconds=2; 8.01~14.00 seconds=3; >14.00 seconds=4 |
| Total number of distractions | no distraction=0; one time=1; two times=2; three or more times=3 |
| **For mobile phone use** |  |
| Total duration of distraction | no distraction=0; 0.01~4.00 seconds=1; 4.01~8.00 seconds=2; 8.01~15.00 seconds=3; >15.00 seconds=4 |
| Proportion of distracted time | no distraction=0; 0.01%~16.67%=1; 16.68%~35.29%=2; 35.30%~63.64%=3; >63.64%=4 |
| Duration of the longest distraction time | no distraction=0; 0.01~3.00 seconds=1; 3.01~7.00 seconds=2; 7.01~13.00 seconds=3; >13.00 seconds=4 |
| Total number of distractions | no distraction=0; one time=1; two times=2; three or more times=3 |
| **For talking with other pedestrians** |  |
| Total duration of distraction | no distraction=0; 0.01~5.00 seconds=1; 5.01~10.00 seconds=2; 10.01~17.00 seconds=3; >17.00 seconds=4 |
| Proportion of distracted time | no distraction=0; 0.01%~20.00%=1; 20.01%~40.91%=2; 40.92%~68.18%=3; >68.18%=4 |
| Duration of the longest distraction time | no distraction=0; 0.01~4.00 seconds=1; 4.01~8.00 seconds=2; 8.01~15.00 seconds=3; >15.00 seconds=4 |
| Total number of distractions | no distraction=0; one time=1; two times=2; three or more times=3 |
| **For eating, drinking, or smoking** |  |
| Total duration of distraction | no distraction=0; 0.01~5.00 seconds=1; 5.01~9.00 seconds=2; 9.01~16.00 seconds=3; >16.00 seconds=4 |
| Proportion of distracted time | no distraction=0; 0.01%~19.35%=1; 19.36%~38.89%=2; 38.90%~68.18%=3; >68.18%=4 |
| Duration of the longest distraction time | no distraction=0; 0.01~3.00 seconds=1; 3.01~7.00 seconds=2; 7.01~13.00 seconds=3; >13.00 seconds=4 |
| Total number of distractions | no distraction=0; one time=1; two times=2; three or more times=3 |

Notes: The total duration of distraction, proportion of distracted time, and duration of the longest distraction time were categorized into five groups based on the quartiles (no distraction=0; *P*_0.1_~*P*_25_=1; *P*_25.1_~ *P*_50_=2; *P*_50.1_~ *P*_75_=3; > *P*_75_=4) by type of distraction. Total number of distractions were categorized into four groups based on distribution.

**Table B4** Description of the grouping of four distraction indicators by type of distraction for sensitivity analysis

| **Distraction indicator** | **Assignment** |
| --- | --- |
| **For all distraction** |  |
| Total duration of distraction | by median: no distraction=0; 0.01~9.00 seconds=1; >9.00 seconds=2  by tertiles: no distraction=0; 0.01~6.00 seconds=1; 6.01~13.00 seconds=2; >13.00 seconds=3  by quintile: no distraction=0; 0.01~3.00 seconds=1; 3.01~7.00 seconds=2; 7.01~12.00 seconds=3; 12.01~18.00 seconds=4; >18.00 seconds=5 |
| Proportion of distracted time | by median: no distraction=0; 0.01%~39.13%=1; >39.13%=2  by tertiles: no distraction=0; 0.01%~25.00%=1; 25.01%~56.52%=2; >56.52%=3  by quintile: no distraction=0; 0.01%~14.81%=1; 14.82%~30.00%=2; 30.01%~50.00%=3; 50.01%~73.08%=4; >73.08%=5 |
| Duration of the longest distraction time | by median: no distraction=0; 0.01~8.00 seconds=1; >8.00 seconds=2  by tertiles: no distraction=0; 0.01~5.00 seconds=1; 5.01~12.00 seconds=2; >12.00 seconds=3  by quintile: no distraction=0; 0.01~3.00 seconds=1; 3.01~6.00 seconds=2; 6.01~10.00 seconds=3; 10.01~16.00 seconds=4; >16.00 seconds=5 |
| Total number of distractions | no distraction=0; one time=1; two times and over=2 |
| **For mobile phone use** |  |
| Total duration of distraction | by median: no distraction=0; 0.01~8.00 seconds=1; >8.00 seconds=2  by tertiles: no distraction=0; 0.01~5.00 seconds=1; 5.01~12.00 seconds=2; >12.00 seconds=3  by quintile: no distraction=0; 0.01~3.00 seconds=1; 3.01~6.00 seconds=2; 6.01~10.00 seconds=3; 10.01~16.00 seconds=4; >16.00 seconds=5 |
| Proportion of distracted time | by median: no distraction=0; 0.01%~35.29%=1; >35.29%=2  by tertiles: no distraction=0; 0.01%~22.22%=1; 22.23%~53.33%=2; >53.33%=3  by quintile: no distraction=0; 0.01%~13.33%=1; 13.34%~27.27%=2; 27.28%~45.45%=3; 45.46%~70.59%=4; >70.59%=5 |
| Duration of the longest distraction time | by median: no distraction=0; 0.01~7.00 seconds=1; >7.00 seconds=2  by tertiles: no distraction=0; 0.01~4.00 seconds=1; 4.01~11.00 seconds=2; >11.00 seconds=3  by quintile: no distraction=0; 0.01~3.00 seconds=1; 3.01~6.00 seconds=2; 6.01~9.00 seconds=3; 9.01~15.00 seconds=4; >15.00 seconds=5 |
| Total number of distractions | no distraction=0; one time=1; two times and over=2 |
| **For talking with other pedestrians** |  |
| Total duration of distraction | by median: no distraction=0; 0.01~10.00 seconds=1; >10.00 seconds=2  by tertiles: no distraction=0; 0.01~6.00 seconds=1; 6.01~14.00 seconds=2; >14.00 seconds=3  by quintile: no distraction=0; 0.01~4.00 seconds=1; 4.01~8.00 seconds=2; 8.01~12.00 seconds=3; 12.01~19.00 seconds=4; >19.00 seconds=5 |
| Proportion of distracted time | by median: no distraction=0; 0.01%~40.91%=1; >40.91%=2  by tertiles: no distraction=0; 0.01%~26.32%=1; 26.33~59.09%=2; >59.09%=3  by quintile: no distraction=0; 0.01%~15.63%=1; 15.64%~31.25%=2; 31.26%~50.00%=3; 50.01%~73.91%=4; >73.91%=5 |
| Duration of the longest distraction time | by median: no distraction=0; 0.01~8.00 seconds=1; >8.00 seconds=2  by tertiles: no distraction=0; 0.01~5.00 seconds=1; 5.01~12.00 seconds=2; >12.00 seconds=3  by quintile: no distraction=0; 0.01~3.00 seconds=1; 3.01~6.00 seconds=2; 6.01~11.00 seconds=3; 11.01~17.00 seconds=4; >17.00 seconds=5 |
| Total number of distractions | no distraction=0; one time=1; two times and over=2 |
| **For eating, drinking, or smoking** |  |
| Total duration of distraction | by median: no distraction=0; 0.01~9.00 seconds=1; >9.00 seconds=2  by tertiles: no distraction=0; 0.01~6.00 seconds=1; 6.01~13.00 seconds=2; >13.00 seconds=3  by quintile: no distraction=0; 0.01~4.00 seconds=1; 4.01~7.00 seconds=2; 7.01~11.00 seconds=3; 11.01~18.00 seconds=4; >18.00 seconds=5 |
| Proportion of distracted time | by median: no distraction=0; 0.01%~38.89%=1; >38.89%=2  by tertiles: no distraction=0; 0.01%~25.00%=1; 25.01%~57.14%=2; >57.14%=3  by quintile: no distraction=0; 0.01%~16.67%=1; 16.68%~30.77%=2; 30.78%~50.00%=3; 50.01%~76.19%=4; >76.19%=5 |
| Duration of the longest distraction time | by median: no distraction=0; 0.01~7.00 seconds=1; >7.00 seconds=2  by tertiles: no distraction=0; 0.01~4.00 seconds=1; 4.01~11.00 seconds=2; >11.00 seconds=3  by quintile: no distraction=0; 0.01~3.00 seconds=1; 3.01~5.00 seconds=2; 5.01~9.00 seconds=3; 9.01~15.00 seconds=4; >15.00 seconds=5 |
| Total number of distractions | no distraction=0; one time=1; two times and over=2 |

Notes: The total duration of distraction, proportion of distracted time, and single longest duration of distraction time for all distracted pedestrians by type of distraction were grouped by median, tertiles, and quintiles, respectively; the total number of distractions for distracted pedestrians were classified as no distraction, one duration of distraction, and two or more durations of distraction. Scores for all four indicators were assigned zero for pedestrians with no distraction.

**Table B5** Sensitivity analyses for discriminant validity of the four distraction indicators by alternating the classification of distraction indicators, all walking distractions combined

| **Safety measure/distraction indicator** | **Harrell’s C statistic in base model^a^** | **Harrell’s C statistic in new model^b^** | **Change in Harrell’s C statistic** |
| --- | --- | --- | --- |
| **For near-crash incidence** |  |  |  |
| Total duration of distraction |  |  |  |
| Two groups by median | 0.5987 (0.5912, 0.6063) | 0.6274 (0.6200, 0.6348) | 0.0286 (0.0113, 0.0461)^*^ |
| Three groups by tertiles | 0.5987 (0.5912, 0.6063) | 0.6287 (0.6213, 0.6361) | 0.0300 (0.0130, 0.0472)^*^ |
| Four groups by quartiles | 0.5987 (0.5912, 0.6063) | 0.6287 (0.6213, 0.6361) | 0.0300 (0.0132, 0.0494)^*^ |
| Five groups by quintiles | 0.5987 (0.5912, 0.6063) | 0.6293 (0.6219, 0.6367) | 0.0305 (0.0141, 0.0502)^*^ |
| Proportion of distracted time |  |  |  |
| Two groups by median | 0.5987 (0.5912, 0.6063) | 0.6271 (0.6197, 0.6345) | 0.0284 (0.0110, 0.0461)^*^ |
| Three groups by tertiles | 0.5987 (0.5912, 0.6063) | 0.6280 (0.6206, 0.6354) | 0.0293 (0.0119, 0.0479)^*^ |
| Four groups by quartiles | 0.5987 (0.5912, 0.6063) | 0.6273 (0.6199, 0.6347) | 0.0285 (0.0121, 0.0473)^*^ |
| Five groups by quintiles | 0.5987 (0.5912, 0.6063) | 0.6278 (0.6204, 0.6352) | 0.0291 (0.0132, 0.0486)^*^ |
| Duration of the longest distraction time |  |  |  |
| Two groups by median | 0.5987 (0.5912, 0.6063) | 0.6283 (0.6209, 0.6357) | 0.0295 (0.0122, 0.0477)^*^ |
| Three groups by tertiles | 0.5987 (0.5912, 0.6063) | 0.6288 (0.6214, 0.6362) | 0.0301 (0.0130, 0.0496)^*^ |
| Four groups by quartiles | 0.5987 (0.5912, 0.6063) | 0.6302 (0.6228, 0.6376) | 0.0315 (0.0137, 0.0508)^*^ |
| Five groups by quintiles | 0.5987 (0.5912, 0.6063) | 0.6284 (0.6210, 0.6358) | 0.0297 (0.0137, 0.0494)^*^ |
| Total number of distractions^c^ |  |  |  |
| Two groups | 0.5987 (0.5912, 0.6063) | 0.6261 (0.6187, 0.6335) | 0.0274 (0.0106, 0.0451)^*^ |
| Three groups | 0.5987 (0.5912, 0.6063) | 0.6261 (0.6187, 0.6335) | 0.0274 (0.0108, 0.0458)^*^ |
| **For frequency of looking left and right** |  |  |  |
| Total duration of distraction |  |  |  |
| Two groups by median | 0.5871 (0.5847, 0.5895) | 0.6164 (0.6141, 0.6187) | 0.0293 (0.0236, 0.0352)^*^ |
| Three groups by tertiles | 0.5871 (0.5847, 0.5895) | 0.6173 (0.6150, 0.6196) | 0.0302 (0.0247, 0.0364)^*^ |
| Four groups by quartiles | 0.5871 (0.5847, 0.5895) | 0.6174 (0.6151, 0.6197) | 0.0303 (0.0247, 0.0366)^*^ |
| Five groups by quintiles | 0.5871 (0.5847, 0.5895) | 0.6177 (0.6154, 0.6201) | 0.0306 (0.0249, 0.0371)^*^ |
| Proportion of distracted time |  |  |  |
| Two groups by median | 0.5871 (0.5847, 0.5895) | 0.6151 (0.6128, 0.6174) | 0.0280 (0.0224, 0.0339)^*^ |
| Three groups by tertiles | 0.5871 (0.5847, 0.5895) | 0.6151 (0.6128, 0.6174) | 0.0280 (0.0225, 0.0341)^*^ |
| Four groups by quartiles | 0.5871 (0.5847, 0.5895) | 0.6154 (0.6131, 0.6177) | 0.0283 (0.0227, 0.0342)^*^ |
| Five groups by quintiles | 0.5871 (0.5847, 0.5895) | 0.6154 (0.6131, 0.6177) | 0.0283 (0.0229, 0.0347)^*^ |
| Duration of the longest distraction time |  |  |  |
| Two groups by median | 0.5871 (0.5847, 0.5895) | 0.6173 (0.6150, 0.6196) | 0.0302 (0.0243, 0.0362)^*^ |
| Three groups by tertiles | 0.5871 (0.5847, 0.5895) | 0.6171 (0.6148, 0.6195) | 0.0300 (0.0245, 0.0361)^*^ |
| Four groups by quartiles | 0.5871 (0.5847, 0.5895) | 0.6181 (0.6158, 0.6204) | 0.0310 (0.0254, 0.0373)^*^ |
| Five groups by quintiles | 0.5871 (0.5847, 0.5895) | 0.6178 (0.6154, 0.6201) | 0.0306 (0.0250, 0.0370)^*^ |
| Total number of distractions^c^ |  |  |  |
| Two groups | 0.5871 (0.5847, 0.5895) | 0.6140 (0.6117, 0.6163) | 0.0269 (0.0214, 0.0327)^*^ |
| Three groups | 0.5871 (0.5847, 0.5895) | 0.6149 (0.6126, 0.6172) | 0.0278 (0.0222, 0.0339)^*^ |
| **For speed crossing the street** |  |  |  |
| Total duration of distraction |  |  |  |
| Two groups by median | 0.5777 (0.5756, 0.5798) | 0.5852 (0.5831, 0.5873) | 0.0075 (0.0041, 0.0107)^*^ |
| Three groups by tertiles | 0.5777 (0.5756, 0.5798) | 0.5869 (0.5849, 0.5890) | 0.0093 (0.0050, 0.0123)^*^ |
| Four groups by quartiles | 0.5777 (0.5756, 0.5798) | 0.5888 (0.5867, 0.5908) | 0.0111 (0.0068, 0.0146)^*^ |
| Five groups by quintiles | 0.5777 (0.5756, 0.5798) | 0.5902 (0.5881, 0.5922) | 0.0125 (0.0080, 0.0159)^*^ |
| Proportion of distracted time |  |  |  |
| Two groups by median | 0.5777 (0.5756, 0.5798) | 0.5807 (0.5786, 0.5829) | 0.0031 (0.0003, 0.0050)^*^ |
| Three groups by tertiles | 0.5777 (0.5756, 0.5798) | 0.5807 (0.5786, 0.5829) | 0.0031 (0.0005, 0.0052)^*^ |
| Four groups by quartiles | 0.5777 (0.5756, 0.5798) | 0.5809 (0.5788, 0.5830) | 0.0033 (0.0007, 0.0053)^*^ |
| Five groups by quintiles | 0.5777 (0.5756, 0.5798) | 0.5812 (0.5791, 0.5833) | 0.0035 (0.0007, 0.0054)^*^ |
| Duration of the longest distraction time |  |  |  |
| Two groups by median | 0.5777 (0.5756, 0.5798) | 0.5847 (0.5826, 0.5867) | 0.0070 (0.0038, 0.0105)^*^ |
| Three groups by tertiles | 0.5777 (0.5756, 0.5798) | 0.5865 (0.5844, 0.5886) | 0.0089 (0.0049, 0.0121)^*^ |
| Four groups by quartiles | 0.5777 (0.5756, 0.5798) | 0.5866 (0.5845, 0.5887) | 0.0089 (0.0050, 0.0121)^*^ |
| Five groups by quintiles | 0.5777 (0.5756, 0.5798) | 0.5880 (0.5859, 0.5901) | 0.0103 (0.0062, 0.0135)^*^ |
| Total number of distractions^c^ |  |  |  |
| Two groups | 0.5777 (0.5756, 0.5798) | 0.5815 (0.5794, 0.5836) | 0.0038 (0.0009, 0.0057)^*^ |
| Three groups | 0.5777 (0.5756, 0.5798) | 0.5815 (0.5794, 0.5836) | 0.0038 (0.0009, 0.0057)^*^ |

Notes:

^a^ Base model: Multivariate regression models including only covariates (i.e., sex, age group, time of day, time of week, violating red light signal, road width, prominent vehicle speed limit sign, median barrier, refuge island).

^b^ New model: Multivariate regression models including all covariates and street-crossing distraction indicator. The link function of multivariate regression was linear function for speed crossing the street, quasi-Poisson function for frequency of looking left and right, and logistic function for near-crash event.

^c^ Total number of distractions for distracted pedestrians was classified into two groups (one time=1; ≥two times=2) and three groups (one time=1; two times=2; ≥three times=3); the total number of distractions was assigned zero for pedestrian with no distraction (Grouping criteria of the four distraction indicators are shown in Table B.3 and Table B.4).

^*^*p*<0.05.

**Table B6** Sensitivity analyses for discriminant validity of four distraction indicators by alternating the grouping of distraction indicator, mobile phone use

| **Safety measure/distraction indicator** | **Harrell’s C statistic in base model^a^** | **Harrell’s C statistic in new model^b^** | **Change in Harrell’s C statistic** |
| --- | --- | --- | --- |
| **For near-crash incidence** |  |  |  |
| Total duration of distraction |  |  |  |
| Two groups by median | 0.6154 (0.6062, 0.6245) | 0.6192 (0.6100, 0.6284) | 0.0039 (-0.0002, 0.0174) |
| Three groups by tertiles | 0.6154 (0.6062, 0.6245) | 0.6197 (0.6105, 0.6289) | 0.0044 (0.0002, 0.0195)^*^ |
| Four groups by quartiles | 0.6154 (0.6062, 0.6245) | 0.6195 (0.6103, 0.6287) | 0.0042 (0.0005, 0.0199)^*^ |
| Five groups by quintiles | 0.6154 (0.6062, 0.6245) | 0.6198 (0.6106, 0.6290) | 0.0044 (0.0012, 0.0217)^*^ |
| Proportion of distracted time |  |  |  |
| Two groups by median | 0.6154 (0.6062, 0.6245) | 0.6188 (0.6096, 0.6280) | 0.0035 (-0.0003, 0.0168) |
| Three groups by tertiles | 0.6154 (0.6062, 0.6245) | 0.6193 (0.6100, 0.6285) | 0.0039 (0.0000, 0.0183)^*^ |
| Four groups by quartiles | 0.6154 (0.6062, 0.6245) | 0.6191 (0.6099, 0.6283) | 0.0037 (0.0004, 0.0192)^*^ |
| Five groups by quintiles | 0.6154 (0.6062, 0.6245) | 0.6209 (0.6116, 0.6301) | 0.0055 (0.0016, 0.0225)^*^ |
| Duration of the longest distraction time |  |  |  |
| Two groups by median | 0.6154 (0.6062, 0.6245) | 0.6197 (0.6105, 0.6289) | 0.0044 (-0.0001, 0.0180) |
| Three groups by tertiles | 0.6154 (0.6062, 0.6245) | 0.6211 (0.6119, 0.6302) | 0.0057 (0.0004, 0.0209)^*^ |
| Four groups by quartiles | 0.6154 (0.6062, 0.6245) | 0.6206 (0.6114, 0.6298) | 0.0053 (0.0007, 0.0202)^*^ |
| Five groups by quintiles | 0.6154 (0.6062, 0.6245) | 0.6202 (0.6110, 0.6294) | 0.0048 (0.0011, 0.0212)^*^ |
| Total number of distractions^c^ |  |  |  |
| Two groups | 0.6154 (0.6062, 0.6245) | 0.6187 (0.6095, 0.6279) | 0.0033 (-0.0004, 0.0160) |
| Three groups | 0.6154 (0.6062, 0.6245) | 0.6184 (0.6092, 0.6276) | 0.0031 (-0.0001, 0.0173) |
| **For frequency of looking left and right** |  |  |  |
| Total duration of distraction |  |  |  |
| Two groups by median | 0.6087 (0.6061, 0.6113) | 0.6200 (0.6174, 0.6226) | 0.0113 (0.0081, 0.0155)^*^ |
| Three groups by tertiles | 0.6087 (0.6061, 0.6113) | 0.6201 (0.6175, 0.6227) | 0.0114 (0.0079, 0.0159)^*^ |
| Four groups by quartiles | 0.6087 (0.6061, 0.6113) | 0.6202 (0.6176, 0.6228) | 0.0115 (0.0081, 0.0158)^*^ |
| Five groups by quintiles | 0.6087 (0.6061, 0.6113) | 0.6200 (0.6174, 0.6226) | 0.0113 (0.0080, 0.0158)^*^ |
| Proportion of distracted time |  |  |  |
| Two groups by median | 0.6087 (0.6061, 0.6113) | 0.6195 (0.6169, 0.6221) | 0.0108 (0.0075, 0.0149)^*^ |
| Three groups by tertiles | 0.6087 (0.6061, 0.6113) | 0.6195 (0.6169, 0.6221) | 0.0108 (0.0074, 0.0151)^*^ |
| Four groups by quartiles | 0.6087 (0.6061, 0.6113) | 0.6195 (0.6169, 0.6221) | 0.0108 (0.0077, 0.0151)^*^ |
| Five groups by quintiles | 0.6087 (0.6061, 0.6113) | 0.6196 (0.6170, 0.6222) | 0.0109 (0.0077, 0.0154)^*^ |
| Duration of the longest distraction time |  |  |  |
| Two groups by median | 0.6087 (0.6061, 0.6113) | 0.6197 (0.6171, 0.6224) | 0.0110 (0.0076, 0.0152)^*^ |
| Three groups by tertiles | 0.6087 (0.6061, 0.6113) | 0.6200 (0.6173, 0.6226) | 0.0112 (0.0079, 0.0154)^*^ |
| Four groups by quartiles | 0.6087 (0.6061, 0.6113) | 0.6200 (0.6174, 0.6227) | 0.0113 (0.0080, 0.0156)^*^ |
| Five groups by quintiles | 0.6087 (0.6061, 0.6113) | 0.6201 (0.6175, 0.6227) | 0.0114 (0.0082, 0.0158)^*^ |
| Total number of distractions^c^ |  |  |  |
| Two groups | 0.6087 (0.6061, 0.6113) | 0.6190 (0.6164, 0.6216) | 0.0103 (0.0069, 0.0144)^*^ |
| Three groups | 0.6087 (0.6061, 0.6113) | 0.6190 (0.6164, 0.6216) | 0.0103 (0.0070, 0.0145)^*^ |
| **For speed crossing the street** |  |  |  |
| Total duration of distraction |  |  |  |
| Two groups by median | 0.5797 (0.5774, 0.5821) | 0.5831 (0.5808, 0.5855) | 0.0034 (0.0008, 0.0058)^*^ |
| Three groups by tertiles | 0.5797 (0.5774, 0.5821) | 0.5835 (0.5812, 0.5859) | 0.0038 (0.0010, 0.0061)^*^ |
| Four groups by quartiles | 0.5797 (0.5774, 0.5821) | 0.5838 (0.5815, 0.5862) | 0.0041 (0.0013, 0.0070)^*^ |
| Five groups by quintiles | 0.5797 (0.5774, 0.5821) | 0.5842 (0.5819, 0.5866) | 0.0045 (0.0017, 0.0075)^*^ |
| Proportion of distracted time |  |  |  |
| Two groups by median | 0.5797 (0.5774, 0.5821) | 0.5805 (0.5782, 0.5829) | 0.0008 (-0.0004, 0.0021) |
| Three groups by tertiles | 0.5797 (0.5774, 0.5821) | 0.5806 (0.5782, 0.5829) | 0.0009 (-0.0003, 0.0024) |
| Four groups by quartiles | 0.5797 (0.5774, 0.5821) | 0.5810 (0.5787, 0.5834) | 0.0013 (-0.0002, 0.0029) |
| Five groups by quintiles | 0.5797 (0.5774, 0.5821) | 0.5807 (0.5784, 0.5831) | 0.0010 (-0.0002, 0.0029) |
| Duration of the longest distraction time |  |  |  |
| Two groups by median | 0.5797 (0.5774, 0.5821) | 0.5830 (0.5807, 0.5853) | 0.0033 (0.0007, 0.0057)^*^ |
| Three groups by tertiles | 0.5797 (0.5774, 0.5821) | 0.5828 (0.5805, 0.5852) | 0.0031 (0.0007, 0.0054)^*^ |
| Four groups by quartiles | 0.5797 (0.5774, 0.5821) | 0.5836 (0.5813, 0.5860) | 0.0039 (0.0014, 0.0065)^*^ |
| Five groups by quintiles | 0.5797 (0.5774, 0.5821) | 0.5838 (0.5814, 0.5861) | 0.0040 (0.0015, 0.0069)^*^ |
| Total number of distractions^c^ |  |  |  |
| Two groups | 0.5797 (0.5774, 0.5821) | 0.5807 (0.5784, 0.5830) | 0.0010 (-0.0003, 0.0025) |
| Three groups | 0.5797 (0.5774, 0.5821) | 0.5807 (0.5784, 0.5831) | 0.0010 (-0.0002, 0.0026) |

Notes:

^a^ Base model: Multivariate regression models including only covariates (i.e., sex, age group, time of day, time of week, violating red light signal, road width, prominent vehicle speed limit sign, median barrier, refuge island).

^b^ New model: Multivariate regression models including all covariates and street-crossing distraction indicator. The link function of multivariate regression was linear function for speed crossing the street, quasi-Poisson function for frequency of looking left and right, and logistic function for near-crash event.

^c^ Total number of distractions for distracted pedestrians was classified into two groups (one time=1; ≥two times=2) and three groups (one time=1; two times=2; ≥three times=3); the total number of distractions was assigned zero for pedestrian with no distraction (Grouping criteria of four distraction indicators are shown in Table B.3 and Table B.4).

^*^*p*<0.05.

**Table B7** Sensitivity analyses for discriminant validity of four distraction indicators by alternating the grouping of distraction indicator, talking with other pedestrians

| **Safety measure/distraction indicator** | **Harrell’s C statistic in base model^a^** | **Harrell’s C statistic in new model^b^** | **Change in Harrell’s C statistic** |
| --- | --- | --- | --- |
| **For near-crash incidence** |  |  |  |
| Total duration of distraction |  |  |  |
| Two groups by median | 0.5976 (0.5893, 0.6059) | 0.6285 (0.6205, 0.6366) | 0.0309 (0.0125, 0.0508)^*^ |
| Three groups by tertiles | 0.5976 (0.5893, 0.6059) | 0.6293 (0.6212, 0.6373) | 0.0316 (0.0127, 0.0518)^*^ |
| Four groups by quartiles | 0.5976 (0.5893, 0.6059) | 0.6300 (0.6220, 0.6381) | 0.0324 (0.0132, 0.0535)^*^ |
| Five groups by quintiles | 0.5976 (0.5893, 0.6059) | 0.6295 (0.6214, 0.6375) | 0.0318 (0.0136, 0.0542)^*^ |
| Proportion of distracted time |  |  |  |
| Two groups by median | 0.5976 (0.5893, 0.6059) | 0.6284 (0.6204, 0.6365) | 0.0308 (0.0124, 0.0504)^*^ |
| Three groups by tertiles | 0.5976 (0.5893, 0.6059) | 0.6286 (0.6205, 0.6366) | 0.0309 (0.0123, 0.0513)^*^ |
| Four groups by quartiles | 0.5976 (0.5893, 0.6059) | 0.6288 (0.6208, 0.6368) | 0.0312 (0.0129, 0.0517)^*^ |
| Five groups by quintiles | 0.5976 (0.5893, 0.6059) | 0.6287 (0.6207, 0.6368) | 0.0311 (0.0142, 0.0520)^*^ |
| Duration of the longest distraction time |  |  |  |
| Two groups by median | 0.5976 (0.5893, 0.6059) | 0.6293 (0.6212, 0.6374) | 0.0317 (0.0128, 0.0506)^*^ |
| Three groups by tertiles | 0.5976 (0.5893, 0.6059) | 0.6292 (0.6212, 0.6373) | 0.0316 (0.0127, 0.0525)^*^ |
| Four groups by quartiles | 0.5976 (0.5893, 0.6059) | 0.6311 (0.6230, 0.6392) | 0.0335 (0.0140, 0.0543)^*^ |
| Five groups by quintiles | 0.5976 (0.5893, 0.6059) | 0.6292 (0.6212, 0.6373) | 0.0316 (0.0137, 0.0532)^*^ |
| Total number of distractions^c^ |  |  |  |
| Two groups | 0.5976 (0.5893, 0.6059) | 0.6282 (0.6202, 0.6363) | 0.0306 (0.0119, 0.0507)^*^ |
| Three groups | 0.5976 (0.5893, 0.6059) | 0.6282 (0.6202, 0.6363) | 0.0306 (0.0123, 0.0512)^*^ |
| **For frequency of looking left and right** |  |  |  |
| Total duration of distraction |  |  |  |
| Two groups by median | 0.5857 (0.5831, 0.5882) | 0.6169 (0.6144, 0.6193) | 0.0312 (0.0243, 0.0382)^*^ |
| Three groups by tertiles | 0.5857 (0.5831, 0.5882) | 0.6179 (0.6154, 0.6204) | 0.0322 (0.0254, 0.0390)^*^ |
| Four groups by quartiles | 0.5857 (0.5831, 0.5882) | 0.6184 (0.6159, 0.6208) | 0.0327 (0.0261, 0.0395)^*^ |
| Five groups by quintiles | 0.5857 (0.5831, 0.5882) | 0.6185 (0.6160, 0.6210) | 0.0328 (0.0260, 0.0400)^*^ |
| Proportion of distracted time |  |  |  |
| Two groups by median | 0.5857 (0.5831, 0.5882) | 0.6158 (0.6133, 0.6183) | 0.0301 (0.0235, 0.0370)^*^ |
| Three groups by tertiles | 0.5857 (0.5831, 0.5882) | 0.6162 (0.6137, 0.6187) | 0.0306 (0.0238, 0.0373)^*^ |
| Four groups by quartiles | 0.5857 (0.5831, 0.5882) | 0.6162 (0.6138, 0.6187) | 0.0306 (0.0238, 0.0376)^*^ |
| Five groups by quintiles | 0.5857 (0.5831, 0.5882) | 0.6165 (0.6140, 0.6189) | 0.0308 (0.0241, 0.0379)^*^ |
| Duration of the longest distraction time |  |  |  |
| Two groups by median | 0.5857 (0.5831, 0.5882) | 0.6177 (0.6152, 0.6202) | 0.0320 (0.0254, 0.0387)^*^ |
| Three groups by tertiles | 0.5857 (0.5831, 0.5882) | 0.6176 (0.6151, 0.6201) | 0.0320 (0.0252, 0.0387)^*^ |
| Four groups by quartiles | 0.5857 (0.5831, 0.5882) | 0.6191 (0.6166, 0.6215) | 0.0334 (0.0266, 0.0403)^*^ |
| Five groups by quintiles | 0.5857 (0.5831, 0.5882) | 0.6186 (0.6161, 0.6210) | 0.0329 (0.0262, 0.0397)^*^ |
| Total number of distractions^c^ |  |  |  |
| Two groups | 0.5857 (0.5831, 0.5882) | 0.6155 (0.6130, 0.6180) | 0.0298 (0.0234, 0.0368)^*^ |
| Three groups | 0.5857 (0.5831, 0.5882) | 0.6162 (0.6138, 0.6187) | 0.0306 (0.0240, 0.0377)^*^ |
| **For speed crossing the street** |  |  |  |
| Total duration of distraction |  |  |  |
| Two groups by median | 0.5797 (0.5775, 0.5820) | 0.5847 (0.5824, 0.5869) | 0.0050 (0.0025, 0.0084)^*^ |
| Three groups by tertiles | 0.5797 (0.5775, 0.5820) | 0.5860 (0.5838, 0.5883) | 0.0063 (0.0033, 0.0097)^*^ |
| Four groups by quartiles | 0.5797 (0.5775, 0.5820) | 0.5875 (0.5852, 0.5897) | 0.0077 (0.0045, 0.0111)^*^ |
| Five groups by quintiles | 0.5797 (0.5775, 0.5820) | 0.5885 (0.5863, 0.5907) | 0.0088 (0.0054, 0.0118)^*^ |
| Proportion of distracted time |  |  |  |
| Two groups by median | 0.5797 (0.5775, 0.5820) | 0.5820 (0.5797, 0.5842) | 0.0022 (0.0001, 0.0039)^*^ |
| Three groups by tertiles | 0.5797 (0.5775, 0.5820) | 0.5819 (0.5797, 0.5842) | 0.0022 (0.0003, 0.0040)^*^ |
| Four groups by quartiles | 0.5797 (0.5775, 0.5820) | 0.5819 (0.5797, 0.5842) | 0.0022 (0.0002, 0.0041)^*^ |
| Five groups by quintiles | 0.5797 (0.5775, 0.5820) | 0.5820 (0.5798, 0.5843) | 0.0023 (0.0003, 0.0041)^*^ |
| Duration of the longest distraction time |  |  |  |
| Two groups by median | 0.5797 (0.5775, 0.5820) | 0.5850 (0.5828, 0.5873) | 0.0053 (0.0027, 0.0088)^*^ |
| Three groups by tertiles | 0.5797 (0.5775, 0.5820) | 0.5857 (0.5835, 0.5880) | 0.0060 (0.0029, 0.0090)^*^ |
| Four groups by quartiles | 0.5797 (0.5775, 0.5820) | 0.5866 (0.5844, 0.5889) | 0.0069 (0.0040, 0.0101)^*^ |
| Five groups by quintiles | 0.5797 (0.5775, 0.5820) | 0.5867 (0.5845, 0.5890) | 0.0070 (0.0038, 0.0103)^*^ |
| Total number of distractions^c^ |  |  |  |
| Two groups | 0.5797 (0.5775, 0.5820) | 0.5821 (0.5798, 0.5844) | 0.0024 (0.0002, 0.0042)^*^ |
| Three groups | 0.5797 (0.5775, 0.5820) | 0.5821 (0.5798, 0.5843) | 0.0023 (-0.0002, 0.0044) |

Notes:

^a^ Base model: Multivariate regression models including only covariates (i.e., sex, age group, time of day, time of week, violating red light signal, road width, prominent vehicle speed limit sign, median barrier, refuge island).

^b^ New model: Multivariate regression models including all covariates and street-crossing distraction indicator. The link function of multivariate regression was linear function for speed crossing the street, quasi-Poisson function for frequency of looking left and right, and logistic function for near-crash event.

^c^ Total number of distractions for distracted pedestrians was classified into two groups (one time=1; ≥two times=2) and three groups (one time=1; two times=2; ≥three times=3); the total number of distractions was assigned zero for pedestrian with no distraction (Grouping criteria of four distraction indicators are shown in Table B.3 and Table B.4).

^*^*p*<0.05.

**Table B8** Sensitivity analyses for discriminant validity of four distraction indicators by changing the grouping of distraction indicator, eating, drinking, or smoking

| **Safety measure/distraction indicator** | **Harrell’s C statistic in base model^a^** | **Harrell’s C statistic in new model^b^** | **Change in Harrell’s C statistic** |
| --- | --- | --- | --- |
| **For near-crash incidence** |  |  |  |
| Total duration of distraction |  |  |  |
| Two groups by median | 0.6154 (0.6057, 0.6251) | 0.6258 (0.6160, 0.6355) | 0.0103 (0.0012, 0.0244)^*^ |
| Three groups by tertiles | 0.6154 (0.6057, 0.6251) | 0.6264 (0.6167, 0.6362) | 0.0110 (0.0018, 0.0250)^*^ |
| Four groups by quartiles | 0.6154 (0.6057, 0.6251) | 0.6273 (0.6175, 0.6370) | 0.0119 (0.0029, 0.0263)^*^ |
| Five groups by quintiles | 0.6154 (0.6057, 0.6251) | 0.6264 (0.6166, 0.6362) | 0.0110 (0.0036, 0.0266)^*^ |
| Proportion of distracted time |  |  |  |
| Two groups by median | 0.6154 (0.6057, 0.6251) | 0.6262 (0.6164, 0.6359) | 0.0107 (0.0011, 0.0248)^*^ |
| Three groups by tertiles | 0.6154 (0.6057, 0.6251) | 0.6266 (0.6169, 0.6364) | 0.0112 (0.0018, 0.0260)^*^ |
| Four groups by quartiles | 0.6154 (0.6057, 0.6251) | 0.6266 (0.6168, 0.6363) | 0.0112 (0.0023, 0.0259)^*^ |
| Five groups by quintiles | 0.6154 (0.6057, 0.6251) | 0.6264 (0.6166, 0.6361) | 0.0110 (0.0026, 0.0262)^*^ |
| Duration of the longest distraction time |  |  |  |
| Two groups by median | 0.6154 (0.6057, 0.6251) | 0.6261 (0.6164, 0.6359) | 0.0107 (0.0010, 0.0249)^*^ |
| Three groups by tertiles | 0.6154 (0.6057, 0.6251) | 0.6270 (0.6172, 0.6367) | 0.0115 (0.0018, 0.0257)^*^ |
| Four groups by quartiles | 0.6154 (0.6057, 0.6251) | 0.6265 (0.6167, 0.6362) | 0.0111 (0.0021, 0.0265)^*^ |
| Five groups by quintiles | 0.6154 (0.6057, 0.6251) | 0.6267 (0.6169, 0.6365) | 0.0113 (0.0037, 0.0276)^*^ |
| Total number of distractions^c^ |  |  |  |
| Two groups | 0.6154 (0.6057, 0.6251) | 0.6255 (0.6158, 0.6352) | 0.0101 (0.0008, 0.0245)^*^ |
| Three groups | 0.6154 (0.6057, 0.6251) | 0.6257 (0.6160, 0.6354) | 0.0103 (0.0014, 0.0249)^*^ |
| **For frequency of looking left and right** |  |  |  |
| Total duration of distraction |  |  |  |
| Two groups by median | 0.6108 (0.6080, 0.6136) | 0.6116 (0.6088, 0.6144) | 0.0008 (0.0000, 0.0022)^*^ |
| Three groups by tertiles | 0.6108 (0.6080, 0.6136) | 0.6117 (0.6089, 0.6145) | 0.0009 (0.0000, 0.0028)^*^ |
| Four groups by quartiles | 0.6108 (0.6080, 0.6136) | 0.6115 (0.6088, 0.6143) | 0.0008 (0.0000, 0.0026)^*^ |
| Five groups by quintiles | 0.6108 (0.6080, 0.6136) | 0.6116 (0.6088, 0.6144) | 0.0008 (0.0001, 0.0029)^*^ |
| Proportion of distracted time |  |  |  |
| Two groups by median | 0.6108 (0.6080, 0.6136) | 0.6115 (0.6087, 0.6143) | 0.0007 (-0.0000, 0.0021) |
| Three groups by tertiles | 0.6108 (0.6080, 0.6136) | 0.6115 (0.6087, 0.6143) | 0.0007 (0.0000, 0.0024)^*^ |
| Four groups by quartiles | 0.6108 (0.6080, 0.6136) | 0.6117 (0.6089, 0.6145) | 0.0009 (0.0001, 0.0026)^*^ |
| Five groups by quintiles | 0.6108 (0.6080, 0.6136) | 0.6116 (0.6089, 0.6144) | 0.0009 (0.0001, 0.0029)^*^ |
| Duration of the longest distraction time |  |  |  |
| Two groups by median | 0.6108 (0.6080, 0.6136) | 0.6116 (0.6088, 0.6144) | 0.0008 (0.0000, 0.0023)^*^ |
| Three groups by tertiles | 0.6108 (0.6080, 0.6136) | 0.6117 (0.6089, 0.6144) | 0.0009 (0.0001, 0.0026)^*^ |
| Four groups by quartiles | 0.6108 (0.6080, 0.6136) | 0.6118 (0.6090, 0.6146) | 0.0010 (0.0002, 0.0028)^*^ |
| Five groups by quintiles | 0.6108 (0.6080, 0.6136) | 0.6117 (0.6090, 0.6145) | 0.0010 (0.0002, 0.0028)^*^ |
| Total number of distractions^c^ |  |  |  |
| Two groups | 0.6108 (0.6080, 0.6136) | 0.6114 (0.6087, 0.6142) | 0.0007 (-0.0000, 0.0021) |
| Three groups | 0.6108 (0.6080, 0.6136) | 0.6120 (0.6093, 0.6148) | 0.0013 (0.0002, 0.0034)^*^ |
| **For speed crossing the street** |  |  |  |
| Total duration of distraction |  |  |  |
| Two groups by median | 0.5814 (0.5789, 0.5839) | 0.5848 (0.5823, 0.5873) | 0.0034 (0.0012, 0.0062)^*^ |
| Three groups by tertiles | 0.5814 (0.5789, 0.5839) | 0.5851 (0.5826, 0.5876) | 0.0037 (0.0013, 0.0064)^*^ |
| Four groups by quartiles | 0.5814 (0.5789, 0.5839) | 0.5851 (0.5826, 0.5876) | 0.0037 (0.0012, 0.0064)^*^ |
| Five groups by quintiles | 0.5814 (0.5789, 0.5839) | 0.5850 (0.5825, 0.5875) | 0.0036 (0.0015, 0.0064)^*^ |
| Proportion of distracted time |  |  |  |
| Two groups by median | 0.5814 (0.5789, 0.5839) | 0.5836 (0.5811, 0.5861) | 0.0022 (0.0003, 0.0049)^*^ |
| Three groups by tertiles | 0.5814 (0.5789, 0.5839) | 0.5836 (0.5811, 0.5861) | 0.0022 (0.0004, 0.0052)^*^ |
| Four groups by quartiles | 0.5814 (0.5789, 0.5839) | 0.5835 (0.5810, 0.5860) | 0.0021 (0.0003, 0.0052)^*^ |
| Five groups by quintiles | 0.5814 (0.5789, 0.5839) | 0.5838 (0.5813, 0.5863) | 0.0024 (0.0005, 0.0055)^*^ |
| Duration of the longest distraction time |  |  |  |
| Two groups by median | 0.5814 (0.5789, 0.5839) | 0.5846 (0.5821, 0.5871) | 0.0032 (0.0011, 0.0058)^*^ |
| Three groups by tertiles | 0.5814 (0.5789, 0.5839) | 0.5850 (0.5825, 0.5875) | 0.0036 (0.0017, 0.0066)^*^ |
| Four groups by quartiles | 0.5814 (0.5789, 0.5839) | 0.5849 (0.5824, 0.5874) | 0.0035 (0.0015, 0.0065)^*^ |
| Five groups by quintiles | 0.5814 (0.5789, 0.5839) | 0.5853 (0.5828, 0.5878) | 0.0039 (0.0017, 0.0068)^*^ |
| Total number of distractions^c^ |  |  |  |
| Two groups | 0.5814 (0.5789, 0.5839) | 0.5838 (0.5813, 0.5863) | 0.0024 (0.0005, 0.0051)^*^ |
| Three groups | 0.5814 (0.5789, 0.5839) | 0.5837 (0.5812, 0.5862) | 0.0023 (0.0005, 0.0054)^*^ |

Notes:

^a^ Base model: Multivariate regression models including only covariates (i.e., sex, age group, time of day, time of week, violating red light signal, road width, prominent vehicle speed limit sign, median barrier, refuge island).

^b^ New model: Multivariate regression models including all covariates and street-crossing distraction indicator. The link function of multivariate regression was linear function for speed crossing the street, quasi-Poisson function for frequency of looking left and right, and logistic function for near-crash event.

^c^ Total number of distractions for distracted pedestrians was classified into two groups (one time=1; ≥two times=2) and three groups (one time=1; two times=2; ≥three times=3); the total number of distractions was assigned zero for pedestrian with no distraction (Grouping criteria of the four distraction indicators are shown in Table B.3 and Table B.4).

^*^*p*<0.05.


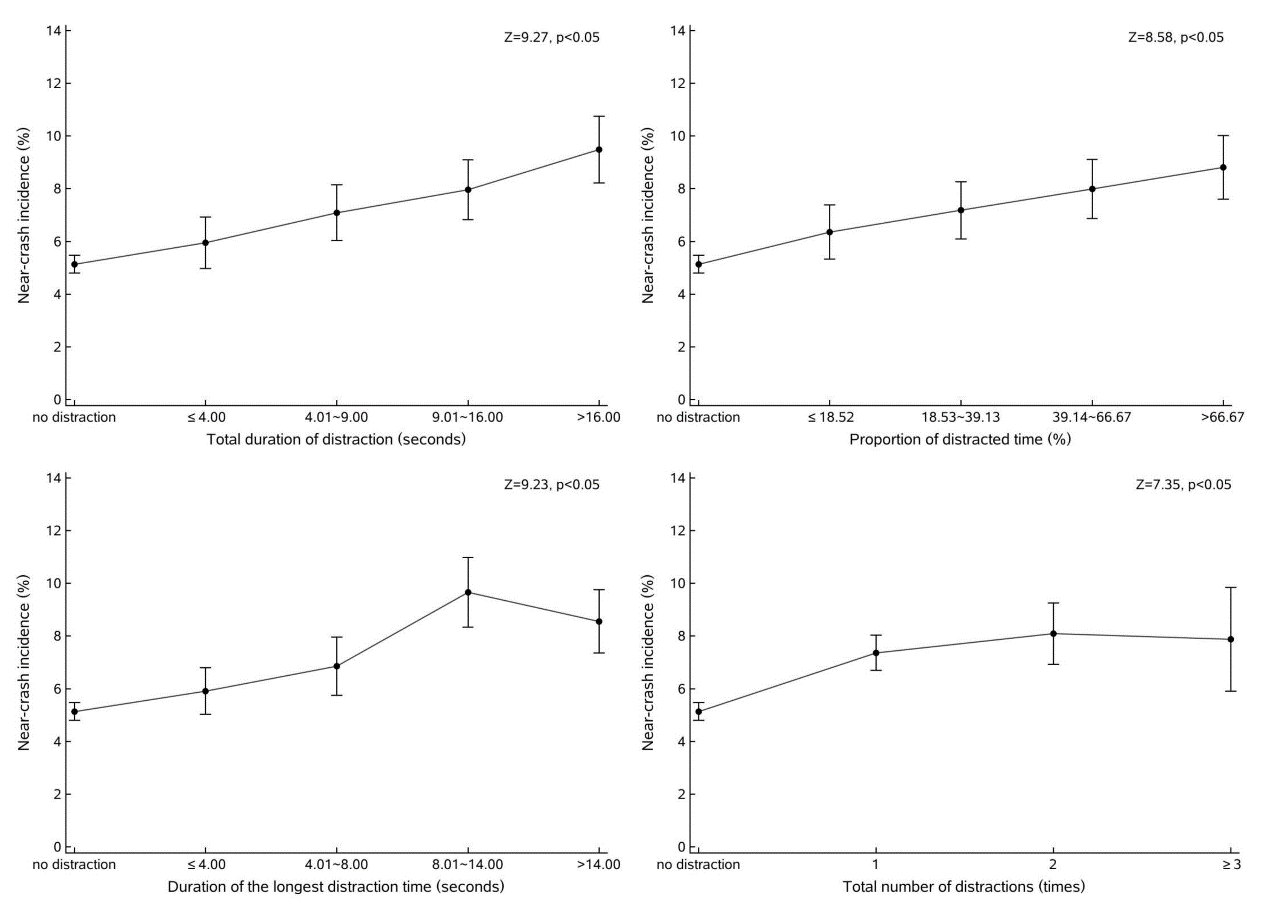


**Fig. B1** Linear graph showing the associations between the four distraction indicators and near-crash incidence, all walking distractions combined

Note: The Cochran-Armitage trend test was used to examine the correlation between the four distraction indicators and near-crash incidence. The first three distraction indicators (total duration of distraction, proportion of distracted time, and duration of the longest distraction time) were categorized into five groups in fitting multivariable models based on the quartiles of sample distributions (no distraction=0; *P*_0.1_~*P*_25_=1; *P*_25.1_~ *P*_50_=2; *P*_50.1_~ *P*_75_=3; > *P*_75_=4) by type of distraction; total number of distractions was categorized into four categories based on distribution (no distraction=0; distracted one time=1; two times=2; three or more times=3).


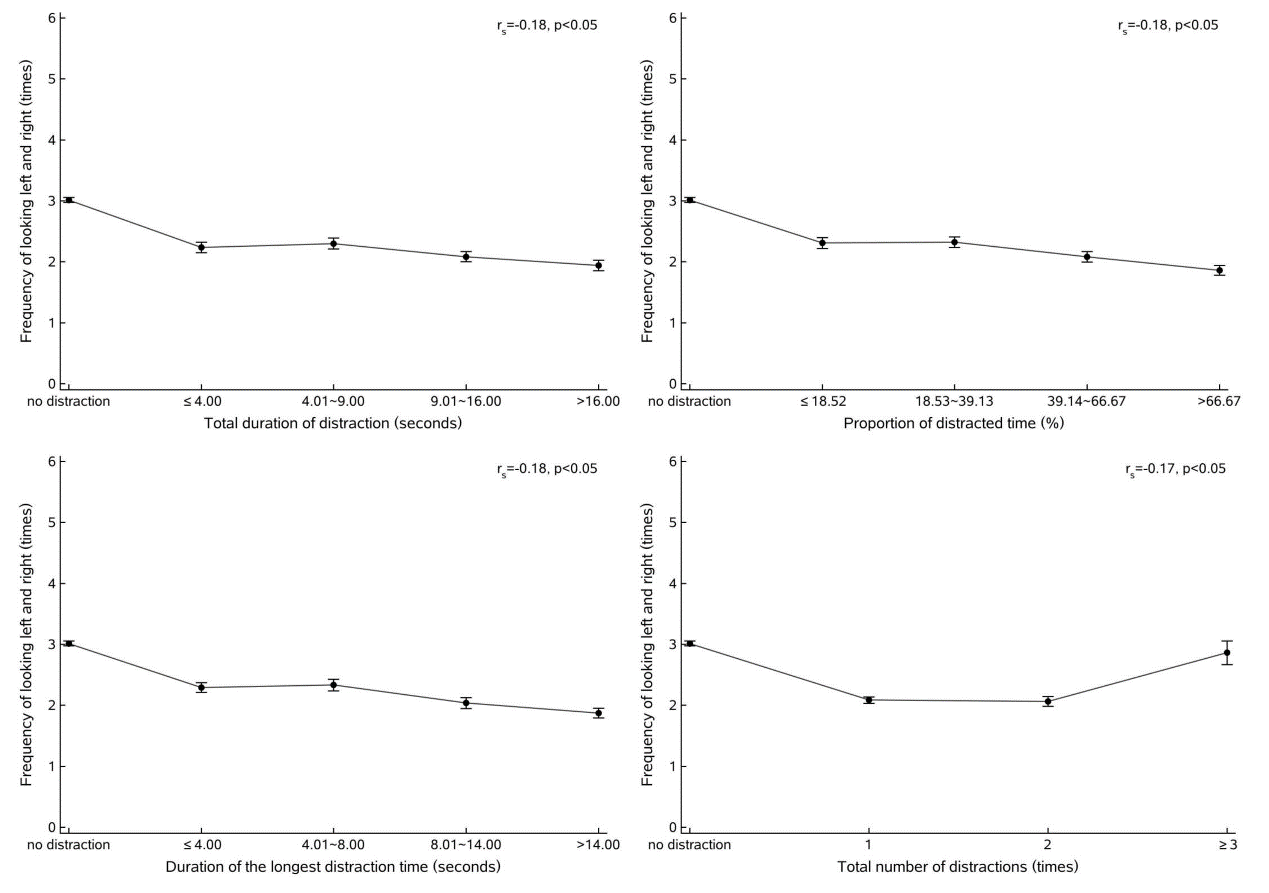


**Fig. B2** Linear graph showing the associations between the four distraction indicators and frequency of looking left and right, all walking distractions combined

Note: The Spearman rank correlation was used to examine the correlation between the four distraction indicators and frequency of looking left and right. The first three distraction indicators (total duration of distraction, proportion of distracted time, and duration of the longest distraction time) were categorized into five groups in fitting multivariable models based on the quartiles of sample distributions (no distraction=0; *P*_0.1_~*P*_25_=1; *P*_25.1_~ *P*_50_=2; *P*_50.1_~ *P*_75_=3; > *P*_75_=4) by type of distraction; total number of distraction was categorized into four categories based on distribution (no distraction=0; distracted one time=1; two times=2; three or more times=3).


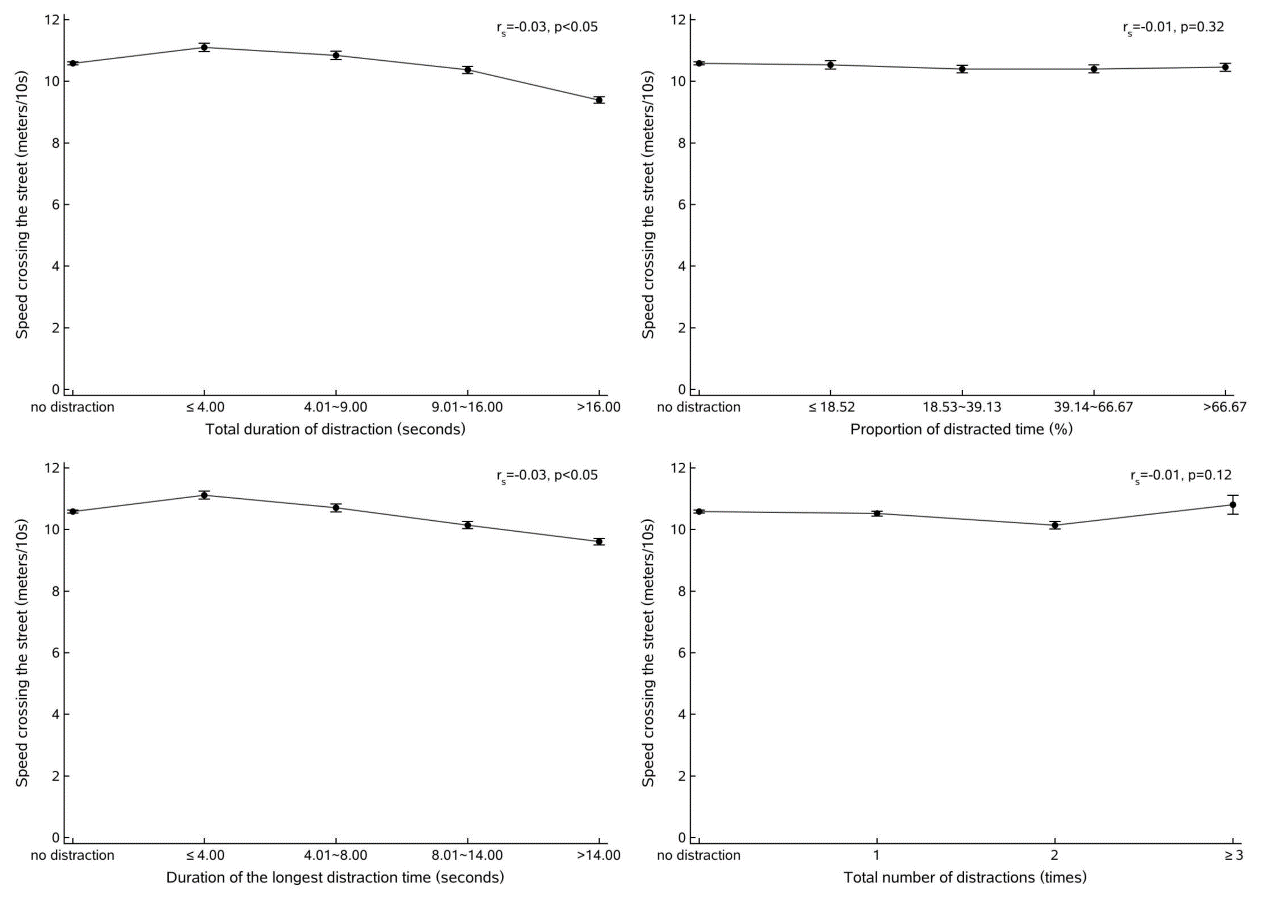


**Fig. B3** Linear graph showing the associations between the four distraction indicators and speed crossing the street, all walking distractions combined

Note: The Spearman rank correlation was used to examine the correlation between the four distraction indicators and speed crossing the street. The first three distraction indicators (total duration of distraction, proportion of distracted time, and duration of the longest distraction time) were categorized into five groups in fitting multivariable models based on the quartiles of sample distributions (no distraction=0; *P*_0.1_~*P*_25_=1; *P*_25.1_~ *P*_50_=2; *P*_50.1_~ *P*_75_=3; > *P*_75_=4) by type of distraction; total number of distraction was categorized into four categories based on distribution (no distraction=0; distracted one time=1; two times=2; three or more times=3).


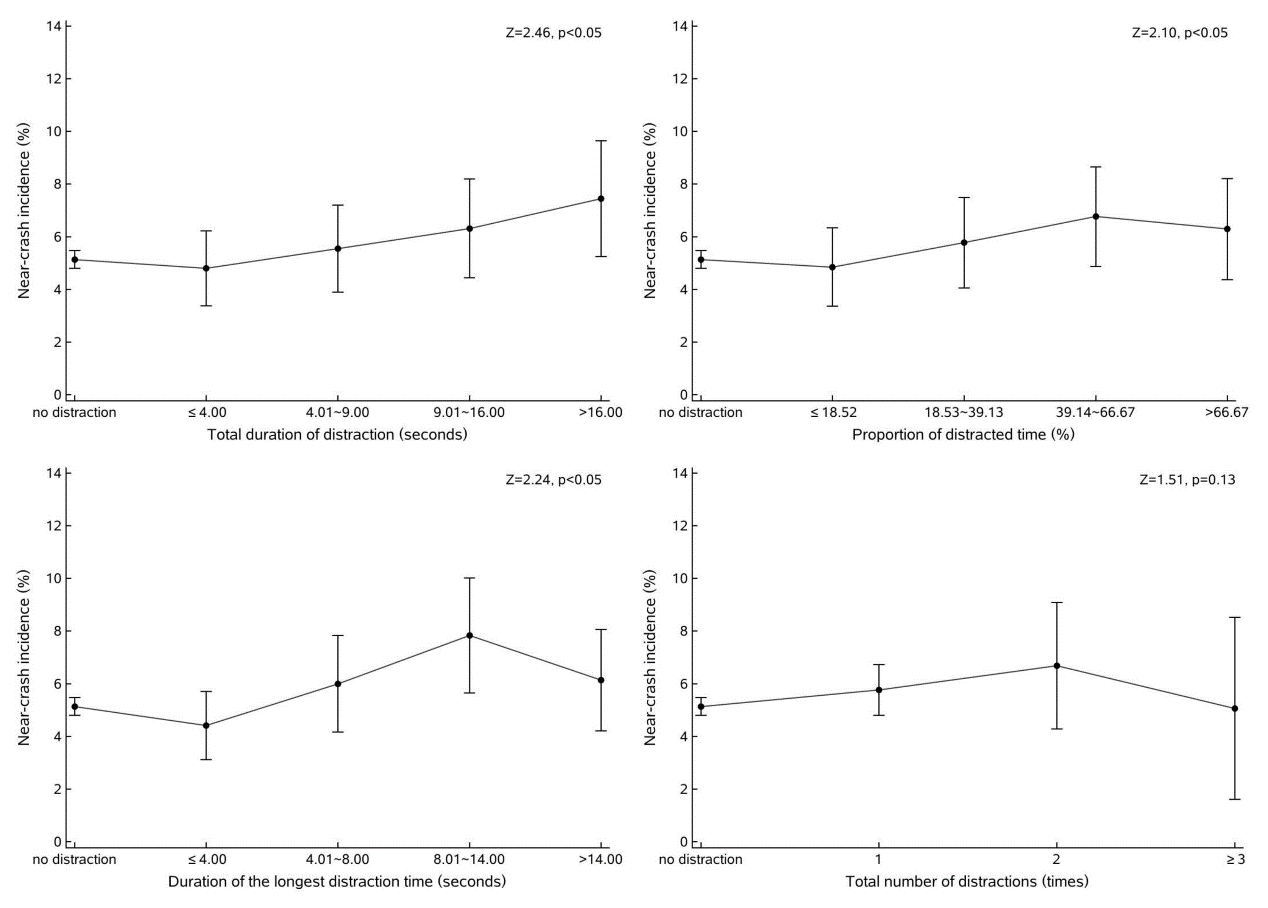


**Fig. B4** Linear graph showing the associations between the four distraction indicators and near-crash incidence, mobile phone use

Note: The Spearman rank correlation was used to exam the correlation between the four distraction indicators and near-crash incidence. The first three distraction indicators (total duration of distraction, proportion of distracted time, and duration of the longest distraction time) were categorized into five groups in fitting multivariable models based on the quartiles of sample distributions (no distraction=0; *P*_0.1_~*P*_25_=1; *P*_25.1_~ *P*_50_=2; *P*_50.1_~ *P*_75_=3; > *P*_75_=4) by type of distraction; total number of distraction was categorized into four categories based on distribution (no distraction=0; distracted one time=1; two times=2; three or more times=3).


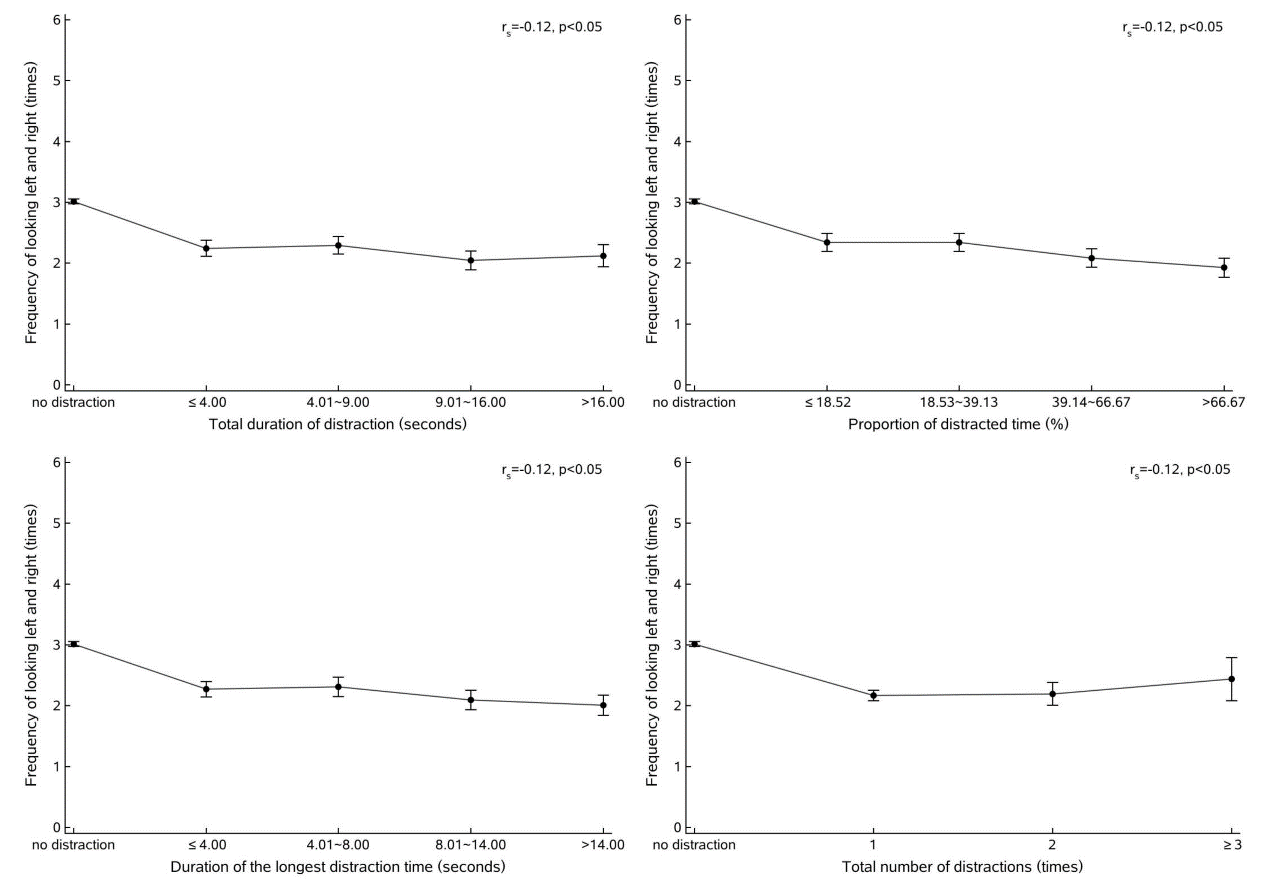


**Fig. B5** Linear graph showing the associations between the four distraction indicators and frequency of looking left and right, mobile phone use

Note: The Spearman rank correlation was used to exam the correlation between the four distraction indicators and frequency of looking left and right. The first three distraction indicators (total duration of distraction, proportion of distracted time, and duration of the longest distraction time) were categorized into five groups in fitting multivariable models based on the quartiles of sample distributions (no distraction=0; *P*_0.1_~*P*_25_=1; *P*_25.1_~ *P*_50_=2; *P*_50.1_~ *P*_75_=3; > *P*_75_=4) by type of distraction; total number of distraction was categorized into four categories based on distribution (no distraction=0; distracted one time=1; two times=2; three or more times=3).


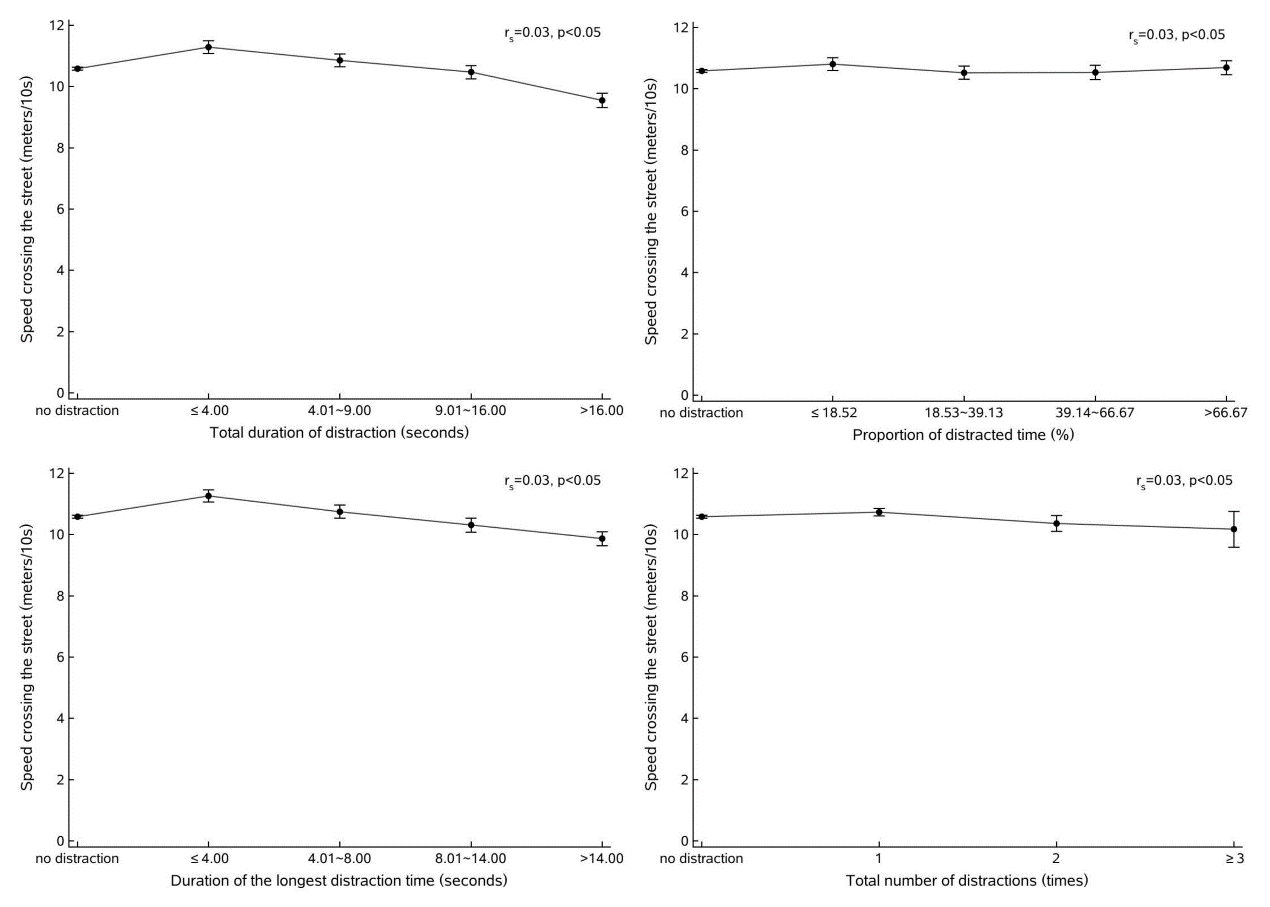


**Fig. B6** Linear graph showing the associations between the four distraction indicators and speed crossing the street, mobile phone use

Note: The Spearman rank correlation was used to exam the correlation between the four distraction indicators and speed crossing the street. The first three distraction indicators (total duration of distraction, proportion of distracted time, and duration of the longest distraction time) were categorized into five groups in fitting multivariable models based on the quartiles of sample distributions (no distraction=0; *P*_0.1_~*P*_25_=1; *P*_25.1_~ *P*_50_=2; *P*_50.1_~ *P*_75_=3; > *P*_75_=4) by type of distraction; total number of distraction was categorized into four categories based on distribution (no distraction=0; distracted one time=1; two times=2; three or more times=3).


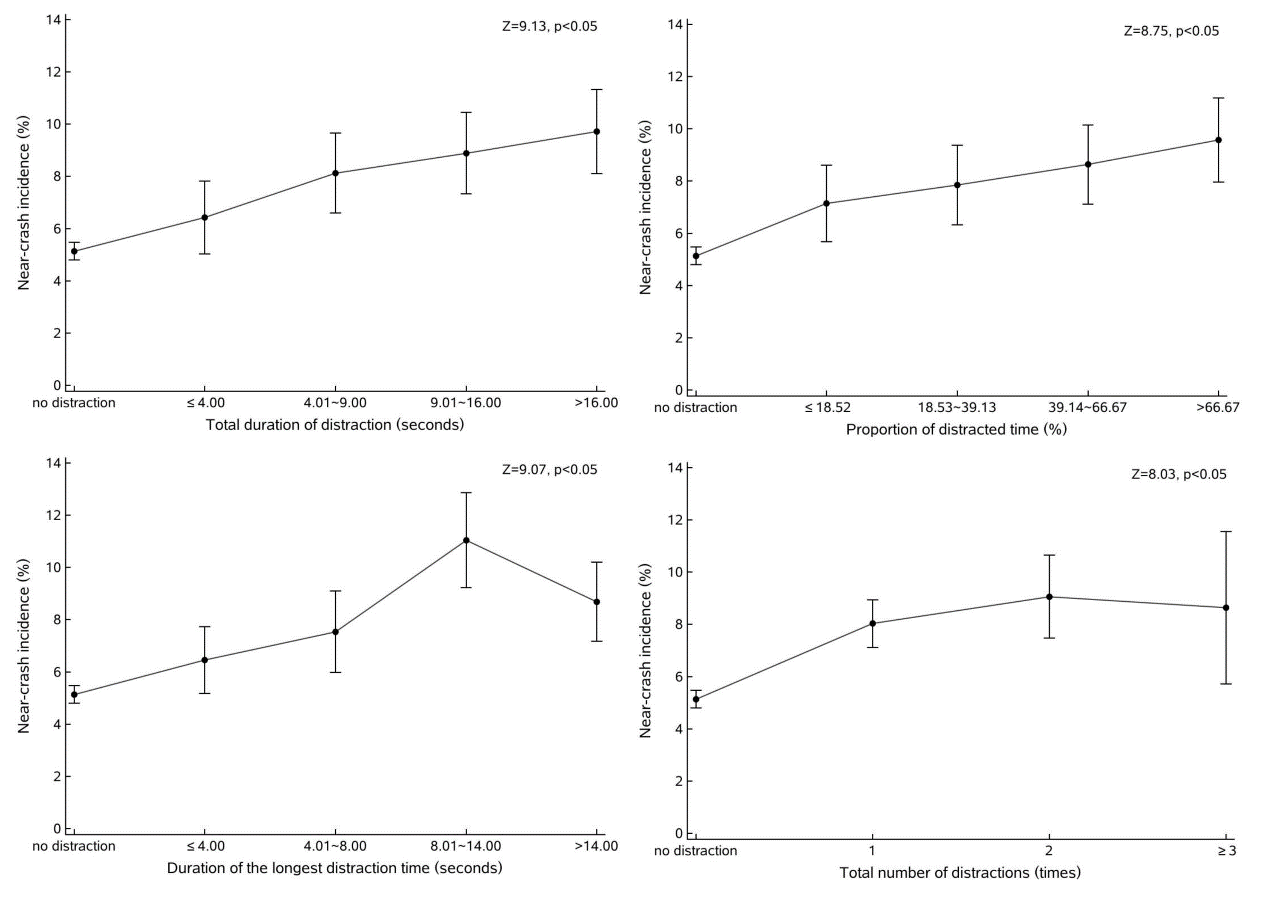


**Fig. B7** Linear graph showing the associations between the four distraction indicators and near-crash incidence, talking with other pedestrians

Note: The Spearman rank correlation was used to exam the correlation between the four distraction indicators and near-crash incidence. The first three distraction indicators (total duration of distraction, proportion of distracted time, and duration of the longest distraction time) were categorized into five groups in fitting multivariable models based on the quartiles of sample distributions (no distraction=0; *P*_0.1_~*P*_25_=1; *P*_25.1_~ *P*_50_=2; *P*_50.1_~ *P*_75_=3; > *P*_75_=4) by type of distraction; total number of distraction was categorized into four categories based on distribution (no distraction=0; distracted one time=1; two times=2; three or more times=3).


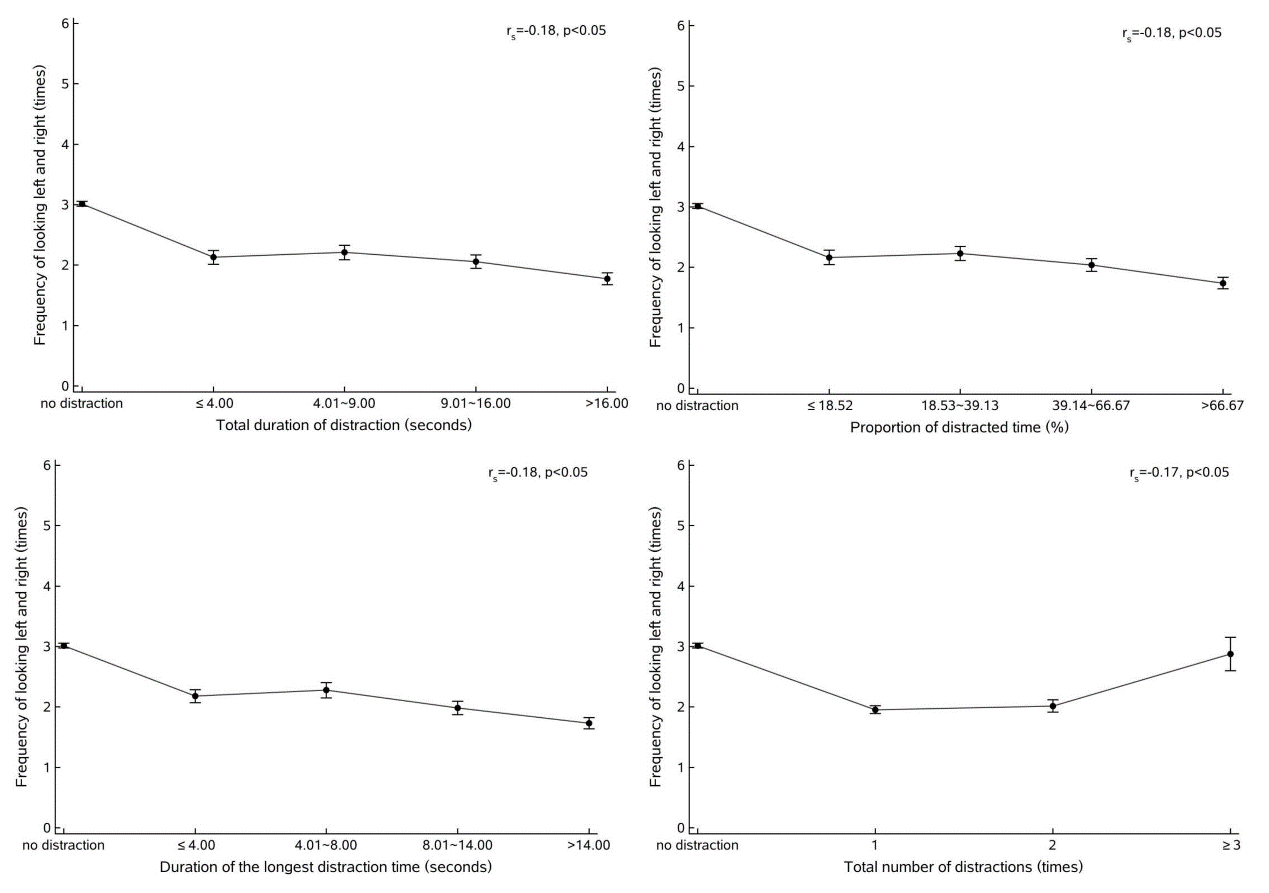


**Fig. B8** Linear graph showing the associations between the four distraction indicators and frequency of looking left and right, talking with other pedestrians

Note: The Spearman rank correlation was used to exam the correlation between the four distraction indicators and s frequency of looking left and right. The first three distraction indicators (total duration of distraction, proportion of distracted time, and duration of the longest distraction time) were categorized into five groups in fitting multivariable models based on the quartiles of sample distributions (no distraction=0; *P*_0.1_~*P*_25_=1; *P*_25.1_~ *P*_50_=2; *P*_50.1_~ *P*_75_=3; > *P*_75_=4) by type of distraction; total number of distraction was categorized into four categories based on distribution (no distraction=0; distracted one time=1; two times=2; three or more times=3).


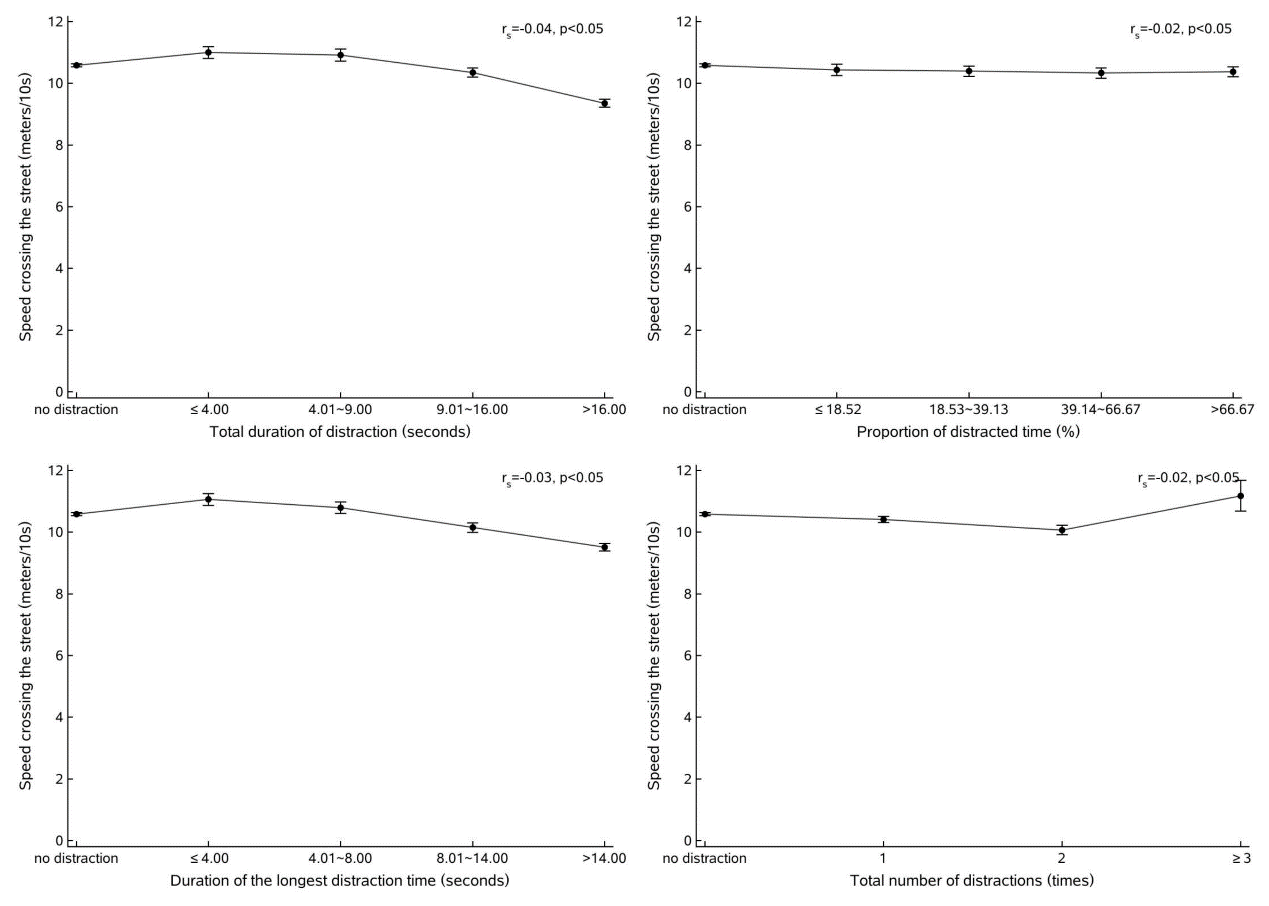


**Fig. B9** Linear graph showing the associations between the four distraction indicators and speed crossing the street, talking with other pedestrians

Note: The Spearman rank correlation was used to exam the correlation between the four distraction indicators and speed crossing the street. The first three distraction indicators (total duration of distraction, proportion of distracted time, and duration of the longest distraction time) were categorized into five groups in fitting multivariable models based on the quartiles of sample distributions (no distraction=0; *P*_0.1_~*P*_25_=1; *P*_25.1_~ *P*_50_=2; *P*_50.1_~ *P*_75_=3; > *P*_75_=4) by type of distraction; total number of distraction was categorized into four categories based on distribution (no distraction=0; distracted one time=1; two times=2; three or more times=3).


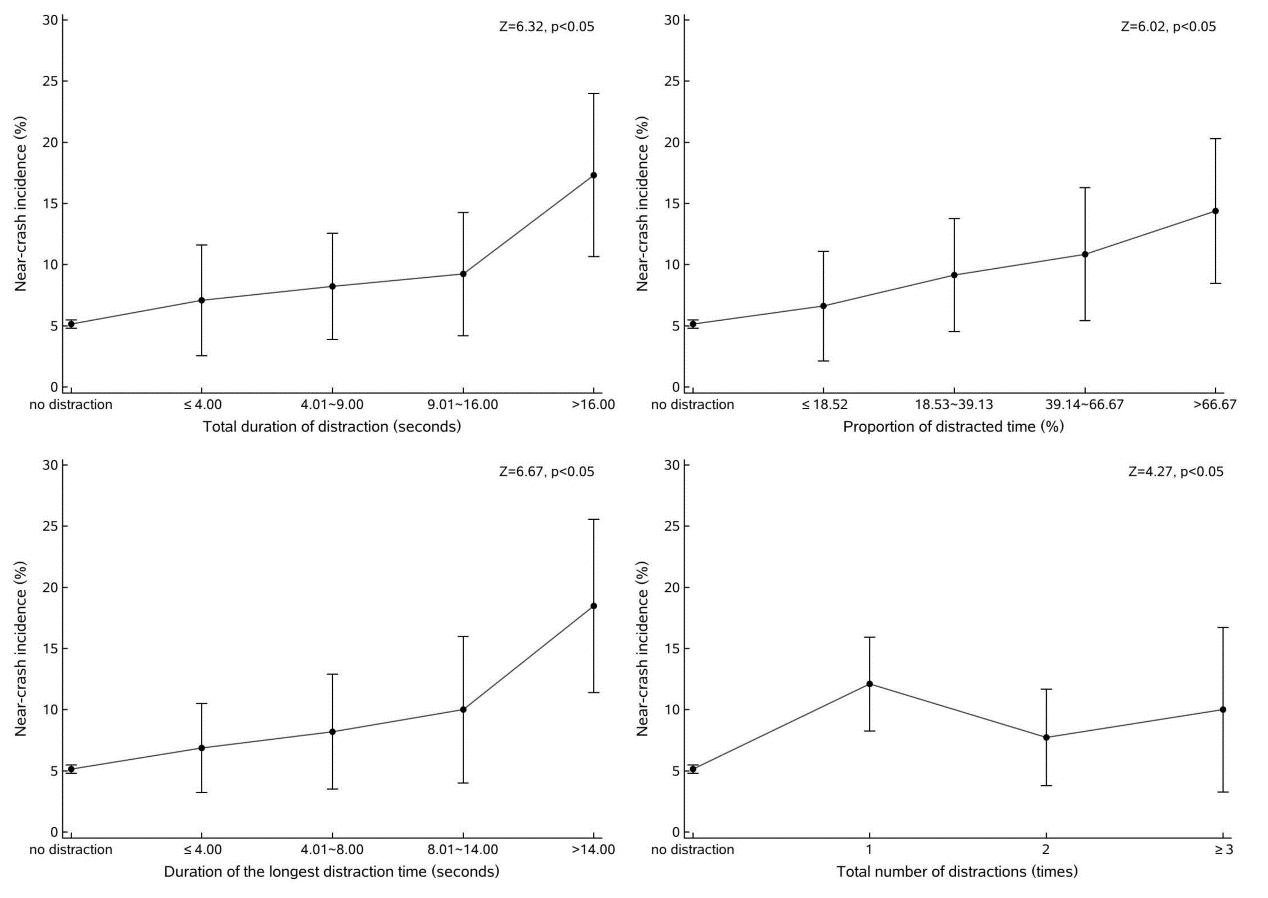


**Fig. B10** Linear graph showing the associations between the four distraction indicators and speed near-crash incidence, eating, drinking, or smoking

Note: The Spearman rank correlation was used to exam the correlation between the four distraction indicators and near-crash incidence. The first three distraction indicators (total duration of distraction, proportion of distracted time, and duration of the longest distraction time) were categorized into five groups in fitting multivariable models based on the quartiles of sample distributions (no distraction=0; *P*_0.1_~*P*_25_=1; *P*_25.1_~ *P*_50_=2; *P*_50.1_~ *P*_75_=3; > *P*_75_=4) by type of distraction; total number of distraction was categorized into four categories based on distribution (no distraction=0; distracted one time=1; two times=2; three or more times=3).


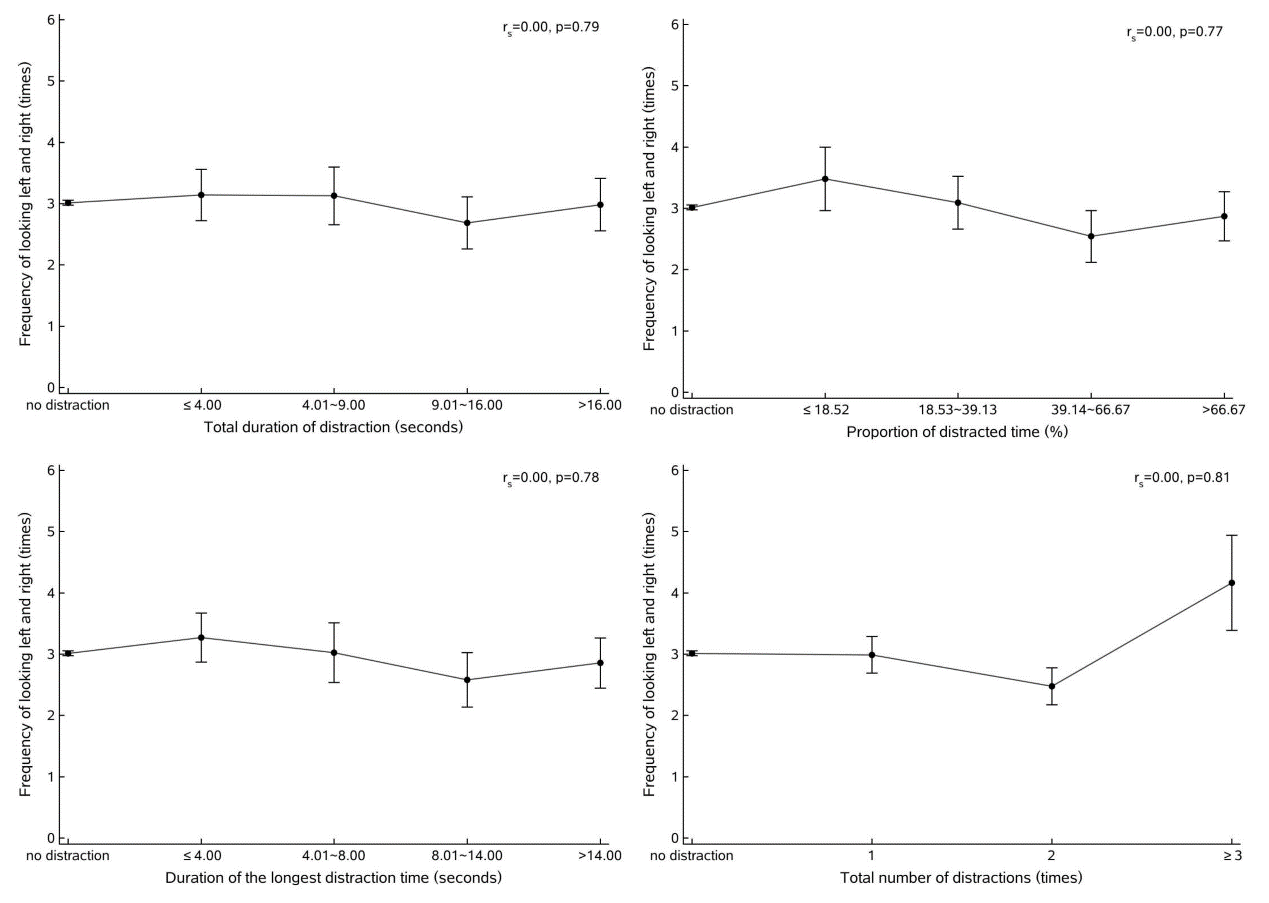


**Fig. B11** Linear graph showing the associations between the four distraction indicators and frequency of looking left and right, eating, drinking, or smoking

Note: The Spearman rank correlation was used to exam the correlation between the four distraction indicators and frequency of looking left and right. The first three distraction indicators (total duration of distraction, proportion of distracted time, and duration of the longest distraction time) were categorized into five groups in fitting multivariable models based on the quartiles of sample distributions (no distraction=0; *P*_0.1_~*P*_25_=1; *P*_25.1_~ *P*_50_=2; *P*_50.1_~ *P*_75_=3; > *P*_75_=4) by type of distraction; total number of distraction was categorized into four categories based on distribution (no distraction=0; distracted one time=1; two times=2; three or more times=3).


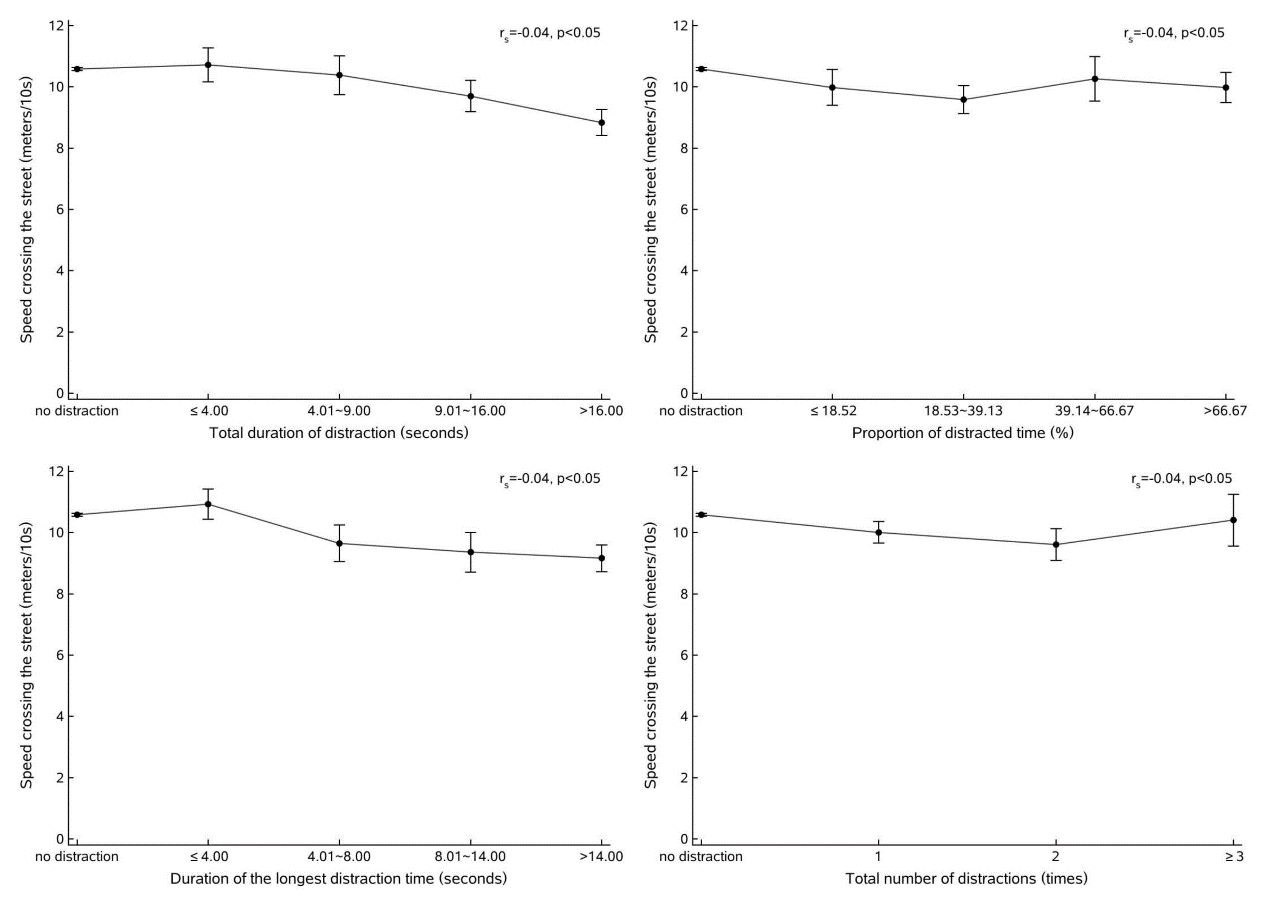


**Fig. B12** Linear graph showing the four distraction indicators and speed crossing the street, eating, drinking, or smoking

Note: The Spearman rank correlation was used to exam the correlation between the four distraction indicators and speed crossing the street. The first three distraction indicators (total duration of distraction, proportion of distracted time, and duration of the longest distraction time) were categorized into five groups in fitting multivariable models based on the quartiles of sample distributions (no distraction=0; *P*_0.1_~*P*_25_=1; *P*_25.1_~ *P*_50_=2; *P*_50.1_~ *P*_75_=3; > *P*_75_=4) by type of distraction; total number of distraction was categorized into four categories based on distribution (no distraction=0; distracted one time=1; two times=2; three or more times=3).
